# Multiple Sustainable Practices Are Crucial for Enhancing the Provisioning of Agroecosystem Services Worldwide

**DOI:** 10.1111/ele.70276

**Published:** 2025-12-01

**Authors:** Luna Medrano, Margarita Ros, Tadeo Sáez‐Sandino, Guiyao Zhou, Dongxue Tao, Kaiyan Zhai, Yue Yin, Tao Zhou, Daniel Revillini, Jose Antonio Pascual, Antonio Rafael Sánchez‐Rodríguez, Raúl Ochoa‐Hueso, María del Mar Alguacil, Daniel Sacristán, Javier Alejandre, Gema del Río, Matthias C. Rilling, Manuel Delgado‐Baquerizo

**Affiliations:** ^1^ Laboratorio de Biodiversidad y Funcionamiento Ecosistémico Instituto de Recursos Naturales y Agrobiología de Sevilla (IRNAS), Consejo Superior de Investigaciones Científicas (CSIC) Sevilla Spain; ^2^ Centro de Edafología y Biología Aplicada del Segura (CEBAS), CSIC Campus Universitario de Espinardo Murcia Spain; ^3^ Hawkesbury Institute for the Environment Western Sydney University Penrith New South Wales Australia; ^4^ State Key Laboratory of Biocontrol, School of Ecology Sun Yat‐sen University Shenzhen China; ^5^ State Key Laboratory of Regional and Urban Ecology, Research Center for Eco‐Environmental Sciences Chinese Academy of Sciences Beijing China; ^6^ School of Ecology and Northeast Asia Biodiversity Research Center Northeast Forestry University Harbin Heilongjiang China; ^7^ Departamento de Biología, Área de Botánica. IVAGRO Universidad de Cádiz Puerto Real Spain; ^8^ Soil Science Unit, Department of Agronomy (DAUCO, ETSIAM) University of Córdoba Córdoba Spain; ^9^ Departamento de Biología Vegetal Universidad de Valencia Valencia Spain; ^10^ Gabinete Técnico Unión de Pequeños Agricultores y Ganaderos Madrid Spain; ^11^ Institute of Biology Freie Universität Berlin Berlin Germany; ^12^ Berlin‐Brandenburg Institute of Advanced Biodiversity Research Berlin Germany

**Keywords:** cereal production, ecosystem services, management combinations, sustainable agriculture, sustainable management practices

## Abstract

Feeding the largest share of the global population, cereal production must enhance sustainability while ensuring food security under global change. Unfortunately, the number of sustainable practices needed to support production, ecosystem services and land conservation remains virtually unknown. We compiled a database of 1570 observations from 349 sites in 57 countries to assess how the number of sustainable practices influences multiple ecosystem services. Our findings reveal that a high number of sustainable practices is crucial for enhancing agroecosystem services such as soil carbon storage, fertility and microbial habitat while supporting yield. Sustainable practices such as crop rotation, limited tillage and incorporation of crop residues were especially important. North America, Eastern Europe and China were particularly dependent on the use of multiple sustainable practices to maintain ecosystem services. Findings underscore the need for integrative strategies employing multiple sustainable practices for mitigating global change, ensuring food security and sustaining ecosystems.

## Graphical abstract

We compiled 1570 observations from 57 countries to assess how the number of sustainable practices affects ecosystem services in cereal systems. We found that multiple practices are essential for improving soil health, yield and ecosystem resilience. Regions like North America, Eastern Europe and China were particularly dependent on multiple sustainable practices, underscoring the global need for integrative strategies to ensure food security and ecological sustainability under global change.

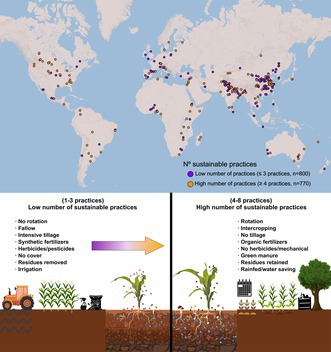

## 1 Introduction

Over the last century, intensive agriculture has significantly increased global food production, increasing our ability to feed a growing human population (Foley et al. 2011). Unfortunately, this system has also resulted in soil degradation and critical losses in soil sustainability (Abdalla et al. 2016; Johnson and Hoyt 1999; Li et al. 2018; Zuber and Villamil 2016). According to the United Nations (UN Chronicle 2021), a third of all soils is already degraded worldwide. Today, we know that agricultural lands are fundamental not only for food security, but also for supporting multiple ecosystem services such as carbon sequestration and soil fertility. Cereal farming constitutes the main source of dietary energy in virtually all populations, contributing about 50% of the dietary energy obtained from food globally (Poutanen et al. 2022). Cereals cover more than 731.6 million hectares of terrestrial surface area (World Bank 2024), making it one of the most extensive agricultural systems globally, but also one of the most stressful on natural resources and degrading to soil health (Foley et al. 2011). Therefore, one of the greatest challenges facing cereal cropping today is ensuring a balance between food security and environmental sustainability. Agriculture worldwide is transitioning toward agroecological practices that enhance soil health and sustainability by reducing tillage, minimizing agrochemicals, retaining crop residues and diversifying rotations (Christel et al. 2021). These practices aim to decrease reliance on external inputs and support soil biodiversity, especially under climate change (Francis and Wezel 2015). While numerous studies demonstrate their environmental benefits (e.g., Jat et al. 2020; Montgomery 2007; Nicholakos et al. 2025), concerns remain about potential yield reductions compared to conventional farming (Seufert et al. 2012). Farmer perceptions and local environmental conditions significantly influence adoption rates (Aghabeygi et al. 2024). Thus, understanding sustainable management strategies is crucial to balance food production and ecosystem protection.

A major question that remains to be answered is how many and what type of sustainable practices are needed to ensure food security while supporting the provision of multiple ecosystem services, and therefore, to best support sustainable agriculture. Although many croplands are labelled as sustainable, their true sustainability is still uncertain for three main reasons. First, evaluating the effects of multiple sustainable practices on agroecosystem services is logistically difficult, as it would require a large number of experimental units. While several recent meta‐analyses have examined cumulative effects of multiple sustainable or conservation practices (e.g., Ejeta and Bai 2025), and other works have synthesised combined impacts of specific agronomic measures (Young et al. 2021), most examine fixed combinations without explicitly testing how the number of practices influences outcomes. Recent theoretical work (e.g., Rillig et al. 2024) highlights the value of quantifying the effect of increasing the number of sustainable practices, but this approach remains untested. Second, existing studies often focus on single practices or single services (e.g., Gebru et al. 2019; Iqbal et al. 2020; Sarker et al. 2023), particularly crop yield, limiting our understanding of broader ecosystem impacts. Finally, there is a need to incorporate diverse environmental and management variables to understand the trade‐offs between productivity and ecosystem service maintenance. By examining several multidimensional management strategies, scientists and farmers can design tailored approaches that enhance cereal production and sustainability (Rillig and Lehmann 2019). With the accumulation of more comprehensive agroecosystem data, it is possible to gain a better understanding of the sustainable management combinations that can promote a decisive advance in sustainable agriculture management globally (Giannarakis et al. 2022). Our research aims to identify the number of sustainable management practices needed to support critical ecosystem services such as crop yield and soil sustainability across wide environmental gradients, and determine if there are standout combinations of practices that outperform others in their combined effects on beneficial ecosystem services, sustainability and agricultural productivity.

**FIGURE 1:**
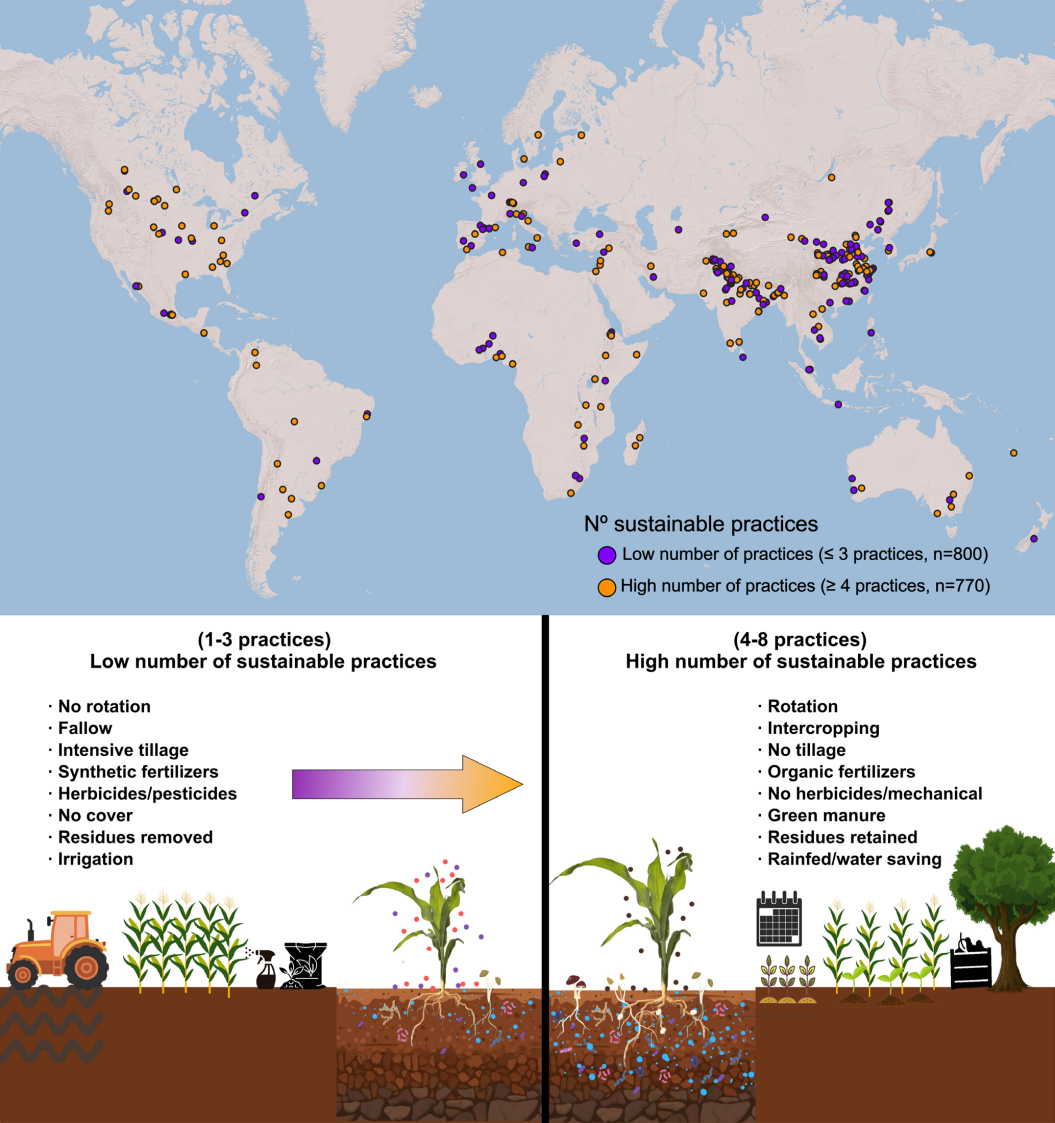
Global distribution of the agricultural studies and diagrammatic representation of the different intensive agricultural management practices and their sustainable counterpart considered in this study. See Table S2 for further information on these treatments.

Here, we assembled a global database including 1570 pairs of observations from 349 locations in 57 countries across all inhabited continents comparing the effects of sustainable vs. intensive practices on ecosystem services (see Tables S1 and S2). This data was collected from 411 agriculture studies at a global scale (Figure 1; see Figure S1). Using this data, we determined the effect of sustainable versus intensive management practices on multiple ecosystem services including soil biodiversity, yield, soil fertility, carbon sequestration, organic matter (OM) decomposition and soil habitat for microbes and microfauna. First, we used a response ratio (i.e., LnRR) to determine the effect of sustainable versus intensive management practices (Table S2) on a wide variety of individual ecosystem attributes associated with multiple ecosystem services (Table S1) in cases of study comparing 1, 2, 3, 4, 5, 6 and +7 sustainable versus intensive practices (see Figure 1, Table S2 for a complete list of management practices). It is important to note that relatively few studies (*n* = 49, 3.12% of studies) incorporated 7 or more sustainable practices, so we merged these cases of studies as +7. We then merged these responses at the ecosystem service level by averaging the responses of all individual ecosystem attributes within the same ecosystem service group and across the number of practices. Our major goal was to assess the relationship between an increasing number of sustainable agricultural practices and the maintenance of vital ecosystem services, such as yield, soil fertility, and carbon sequestration, an approach that allows us to analyze how these practices collectively support enhanced ecosystem health and agricultural productivity on a global scale.

BOX 1 **Calculation of response rations (Osenberg et al. 1999).**

1

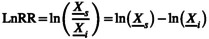

## 2 Methods

### 2.1 Literature Search

We conducted a meta‐analytical search of the literature in November 2023 including all publications available in the Web of Science and Scopus databases. The combination of keywords was: (cropland OR agriculture) AND management AND (wheat OR barley OR maize OR rice) AND (ecosystem function) NOT milk. The combination was used for all the mentioned variable comparisons by replacing the words ecosystem function with the appropriate keywords (carbon, nitrogen, phosphorus, nutrient availability, yield, food production, biomass, biodiversity, microorganism and enzyme activity).

The following criteria were used to select the studies included in the analysis: (1) the studies compared intensive agriculture management with at least one of the selected sustainable agriculture practices, (2) the studies contained sufficient data (more than one response variable) collected for the current types of soil management, (3) the experiment must have been conducted under field conditions and (4) the type of management was not changed during the experiment.

We kept 411 papers included in this meta‐analysis after passing several acceptance criteria (see Appendix S1 for a complete list of publications). The details of the selection of the studies carried out are presented in a PRISMA chart (Figure S1) and references used are listed in the Supporting Information.

### 2.2 Effect of Sustainable Management on Agroecosystem Services

We first identified the corresponding intensive (control) and sustainable treatments in order to calculate the effect sizes of sustainable practices relative to intensive management for each response variable. In particular, we used a response ratio (Box 1) to determine the effect of sustainable versus intensive management (Table S2) on individual ecosystem attributes (Table S1) representing different aspects of biodiversity and ecosystem services, where LnRR is the response ratio, $X_{s}$ and $X_{i}$ are the mean values for a given ecosystem individual attribute under sustainable and intensive management practices, respectively. In all cases, the intensive treatment served as the baseline or reference condition against which the sustainable practice was evaluated. As such, every calculated effect size reflects the magnitude and direction of change resulting specifically from the implementation of a sustainable practice when compared to its intensive counterpart, as defined in each individual study. This consistent within‐study approach ensures that our results isolate the impact of sustainable practices from other confounding factors and allow for meaningful synthesis across a diverse set of experimental conditions. Following these calculations, the mean effects and 95% confidence interval (95% CI) were calculated (see Table S3). The effect of sustainable management was considered to be significant if the 95% CI of the response variable did not overlap zero (Luo et al. 2006). To assess climate influence, we split the dataset into arid (aridity index < 1) and non‐arid (index > 1) regions using the UNEP definition and repeated the analysis by subgroup (see Figure S4). Given the exploratory aim, we did not correct for multiple comparisons, following standard ecological synthesis practices (e.g., Gurevitch et al. 2018) that prioritise trend interpretation over strict hypothesis testing.

After calculating effect sizes for each individual agroecosystem attribute (Table S1), we then aggregated these values to estimate the overall effect of management on each agroecosystem service (six different agroecosystem services: crop yield and soil fertility, carbon sequestration, soil organic matter (SOM) decomposition, habitat and biodiversity; Table S1). This was done by averaging the effect sizes of the variables associated with a given service, allowing us to assess how sustainable practices influence agroecosystem services as integrated outcomes rather than isolated measures. These were defined based on multiple attributes measured in our database (Table S1).

The database contained diverse types of agricultural management practices. Following the effect size computation, and based on the management practices applied in each treatment we classified each individual practice as either sustainable (coded as 1) or intensive (coded as 0), according to the criteria detailed in Table S2. This binary classification enabled the quantification of the total number of sustainable practices implemented per treatment, which was calculated by summing the binary values assigned to each management practice. This approach provided a straightforward and standardized metric for quantifying the degree of sustainability in each treatment across studies.

Across the dataset, the number of simultaneously applied sustainable practices ranged from 1 to 8. Studies including 7 and 8 practices were merged given the lack of studies including 8 sustainable practices simultaneously. In this way, it was possible to identify an optimal number of interventions, both biotic and abiotic to achieve high biodiversity and agricultural ecosystem functions. The responses of cases of study including 7–8 sustainable versus intensive management practices were grouped together due to the relatively low existing data. For some of the analyses in this paper, when comparing a low versus a high number of management practices, we divided our database into two equal groups of categories including low (1–3 practices) and high (4–8 practices) numbers of agricultural practices. This division was made considering the potential impact of the practices on the outcomes studied, making sure to balance the number of studies included in each group. Therefore, we aimed to ensure a representative comparison between the two categories, allowing us to fairly assess the effects associated with different levels of agricultural practices and to compare whether there are different relationships between environmental variables and ecosystem service responses.

### 2.3 Environmental Conditions

Information on latitude, longitude, elevation, aridity index (Mean Annual Precipitation/Evapotranspiration meaning more water availability), Mean Annual Temperature (MAT), Mean Diurnal Range (MDR), Temperature Seasonality (TSEA) and Precipitation Seasonality (PSEA) were also compiled. Latitude, longitude and elevation were taken directly from the original study. The climate data were extracted from the Worldclim database (https://www.worldclim.org/) using geographical information. Soil pH, sand content, SOC and phosphorus data (Total P and Labile Available P) were obtained from the SoilGrids database (https://www.isric.org/explore/soilgrids). Plant cover, which corresponds to the fraction of ground covered by green vegetation, was obtained from Copernicus Global Land Service (Buchhorn et al. 2020). The Normalised Difference Vegetation Index (NDVI index), a reliable proxy for above‐ground net primary production, was obtained from the MODIS/Terra MOD13Q1 Version 6 product at 250‐m spatial resolution. Moreover, Leaf Area Index (LAI) was also obtained from the MODIS/Terra MOD15A2H at 500‐m spatial resolution.

Environmental variables were extracted using QGIS 3.38.0 by applying a 5 km buffer around each study's latitude and longitude, averaging all raster pixels within this area to better capture local conditions. This approach accounts for spatial uncertainty and matches typical field trial sizes (1–10 ha), ensuring reliable environmental estimates.

### 2.4 Correlation Between Environmental Factors and the Effect of an Increasing Number of Sustainable Managements on Agroecosystem Services

Variance partitioning modelling was used to quantify the contribution of ecological factors in explaining the agriculture sustainable management effect. In particular, this analysis allows us to determine if spatial, climatic and soil variables can explain a unique part of the variance that is not explained by the effect of sustainable management in cropping systems. The variance partitioning linked to spatial variables (latitude, elevation, slope), climate variables (aridity index, MAP, MDR, TSEA, PSEA), soil properties (soil pH, sand content, SOC, total P) and plant properties (NDVI, LAI, plant cover) on agroecosystem services (crop yield and soil fertility, carbon sequestration, SOM decomposition, habitat and biodiversity) was calculated using the *varpart* function from the vegan package of R statistical software (Oksanen 2015). To further evaluate the contribution of different groups of explanatory variables on yield variation, separate redundancy analyses (RDA) were performed for all variables. The statistical significance and proportion of explained variance (*R* ^2^) for each group are reported in Table S4.

Elevation and slope data were sourced from the Advanced Land Observation Satellite (ALOS) (Hamazaki 1999). Climate variables were derived from the Worldclim database version 2 (Fick and Hijmans 2017). The aridity index was calculated using the Global Aridity Index and Potential Evapotranspiration (ET0) Database (Zomer et al. 2022). Information regarding plant cover, which refers to the proportion of ground area covered by green vegetation, was retrieved from the Copernicus Global Land Service (Buchhorn et al. 2020). Fine texture, defined as the percentage of clay and silt, along with soil organic carbon and soil pH, was obtained from the SoilGrids database (https://soilgrids.org/), whereas soil total phosphorus data were sourced from Global Gridded Soil Phosphorus Distribution Maps at a resolution of 0.5 degrees (Yang et al. 2014). The NDVI index, which serves as an effective proxy for above‐ground net primary production, was collected from the MODIS/Terra MOD13Q1 Version 6 product, having a spatial resolution of 250 m. Additionally, Leaf Area Index (LAI) data were derived from the MODIS/Terra MOD15A2H dataset at a resolution of 500 m.

We also used Spearman correlation to further investigate the relationships between the above ecological drivers and the responses of agroecosystem services to sustainable agricultural management. Correlation analysis was conducted in SPSS 26.0 (IBM).

### 2.5 Mapping the Global Distribution of Sustainable Management Effects

To predict the extent of the effect of sustainable management with a low and high number of sustainable practices on soil fertility and carbon sequestration at the global scale, we used Random Forest regression analysis (Breiman 2001) with several environmental variables: elevation, slope, MAT, MDR, PSEA, TSEA, aridity index, fine texture, soil pH, soil organic carbon (SOC), soil total phosphorus, plant cover, NDVI and Leaf area index (LAI). To ensure consistency, input datasets with varying spatial resolutions were standardized before modeling. High‐resolution rasters were aggregated using the aggregate() function in R to compute mean values in coarser cells, then all layers were resampled to a common 25 km resolution with the resample() function. This preprocessing minimized uncertainty from resolution mismatches in the random forest model.

We used the R package “randomForest” to build a Random Forest model. Our model configuration consisted of 999 decision trees and conducted 100 replicates to identify the most robust combinations for predicting the effect of sustainable management. To evaluate the accuracy of our predictions, we generated a random forest model (the importance and statistical significance of each predictor were computed using the *rfPermute* package of R statistical software). To provide a visualisation of the most decisive predictions we applied a mask and calculated the Mahalanobis distance for a multidimensional point relative to the locations that were used in the model. The Mahalanobis distance was used to create a spatial mask highlighting areas dissimilar to the training data. This approach improves model robustness by avoiding extrapolation into poorly represented environments without excluding data. It is widely used in global modelling for effective outlier management (e.g., Feng et al. 2022; Guirado et al. 2023). Areas beyond the 0.95 quantile of the chi‐square distribution with six degrees of freedom were flagged and masked (Mallavan et al. 2010). The modelling approach was then validated by returning the predicted values (*x* axis) versus the observed values (*y* axis) (Piñeiro et al. 2008). The data are standardised between 0 and 1 for both low and high practice scenarios, allowing for clearer comparisons and insights into the effectiveness of sustainable management strategies across diverse environmental contexts. This approach was also used by Guirado et al. (2022) to identify outliers at a global scale.

To avoid collinearity between variables in correlation and random forest analysis, the variance inflation factor (VIF) was calculated to ensure that our explanatory variables do not suffer from collinearity (a value > 5 would indicate a high correlation and, thus, multicollinearity; see Table S5). We identified collinearity between total phosphorus (P) and available P. We selected total P for further analysis.

## 3 Results

### 3.1 Multiple Management Practices Are Needed to Support Sustainable Agriculture

**FIGURE 2:**
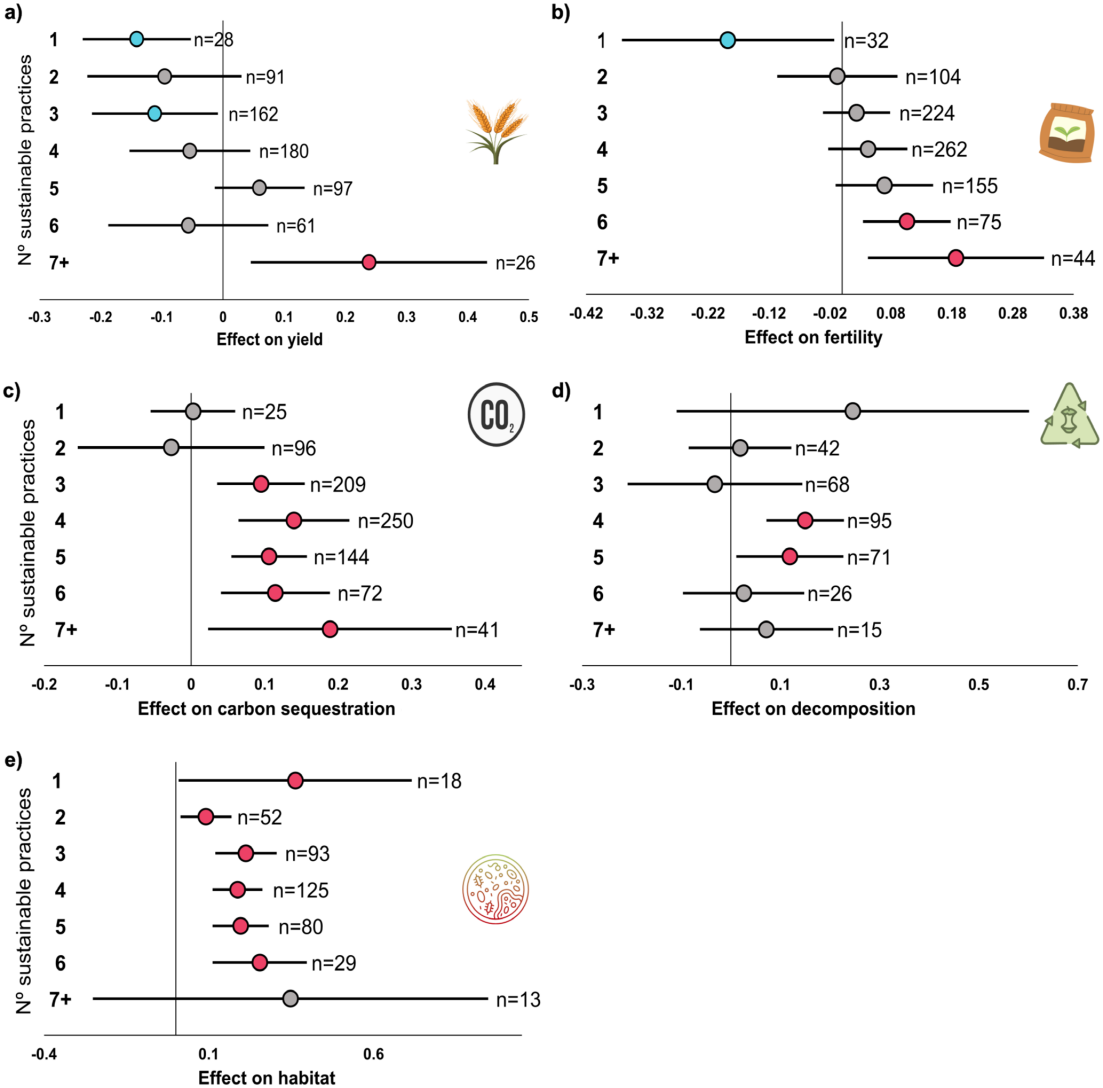
Effect (lnRR) of multiple sustainable agriculture versus intensive agriculture applying a different number of sustainable practices on (a) crop yield; (b) soil fertility; (c) carbon sequestration; (d) SOM decomposition and (e) soil habitat. The error bars show 95% confidence intervals (CI), coloured points indicate significant difference at *p*‐value < 0.05, significantly positive in red and significantly negative in blue. Non‐significant changes are denoted by grey points. Numbers (*n*) indicate the number of studies.

We first compiled a global dataset (Figure 1; Figure S1) and assessed the link between agroecosystem services (crop yield and carbon sequestration, soil fertility, habitat and biodiversity, Table S1; Figure 2; Figure S2) and the application of sustainable agricultural management and the number of implemented practices. Our results provide direct evidence to support the conclusion that when more sustainable agricultural practices are implemented in crop fields, more positive effects will be obtained via the increased provision of multiple agroecosystem services. More specifically, we found that increasing the number of sustainable management practices simultaneously increased crop yield, soil fertility, carbon sequestration, OM decomposition, and habitat (Figure 2). Yield responded negatively when only a single practice was applied, but showed a significant positive effect when seven or more practices were implemented (Figure 2a), though this category included relatively few studies (*n* = 49) and should be interpreted cautiously (Table S3). Similarly, the implementation of a greater number of sustainable agricultural practices (at least 3) also helped build soil carbon stocks, significantly improving carbon sequestration (Figure 2c). Soil fertility showed a clear positive gradient, with significant effects emerging from six practices onwards (Figure 2b). Finally, although less common in our study, a small number of sustainable practices can positively impact key soil aspects, including habitat provision for diverse soil organisms like bacteria, fungi, and microfauna (Figure 2e). We also observed potential positive effects on soil biodiversity, although the number of studies applying multiple practices simultaneously was limited, leading to greater uncertainty in this result (Figure S2).

### 3.2 Environmental Context Fine‐Tunes the Effects of Multiple Sustainable Practices on Agroecosystem Services

**FIGURE 3:**
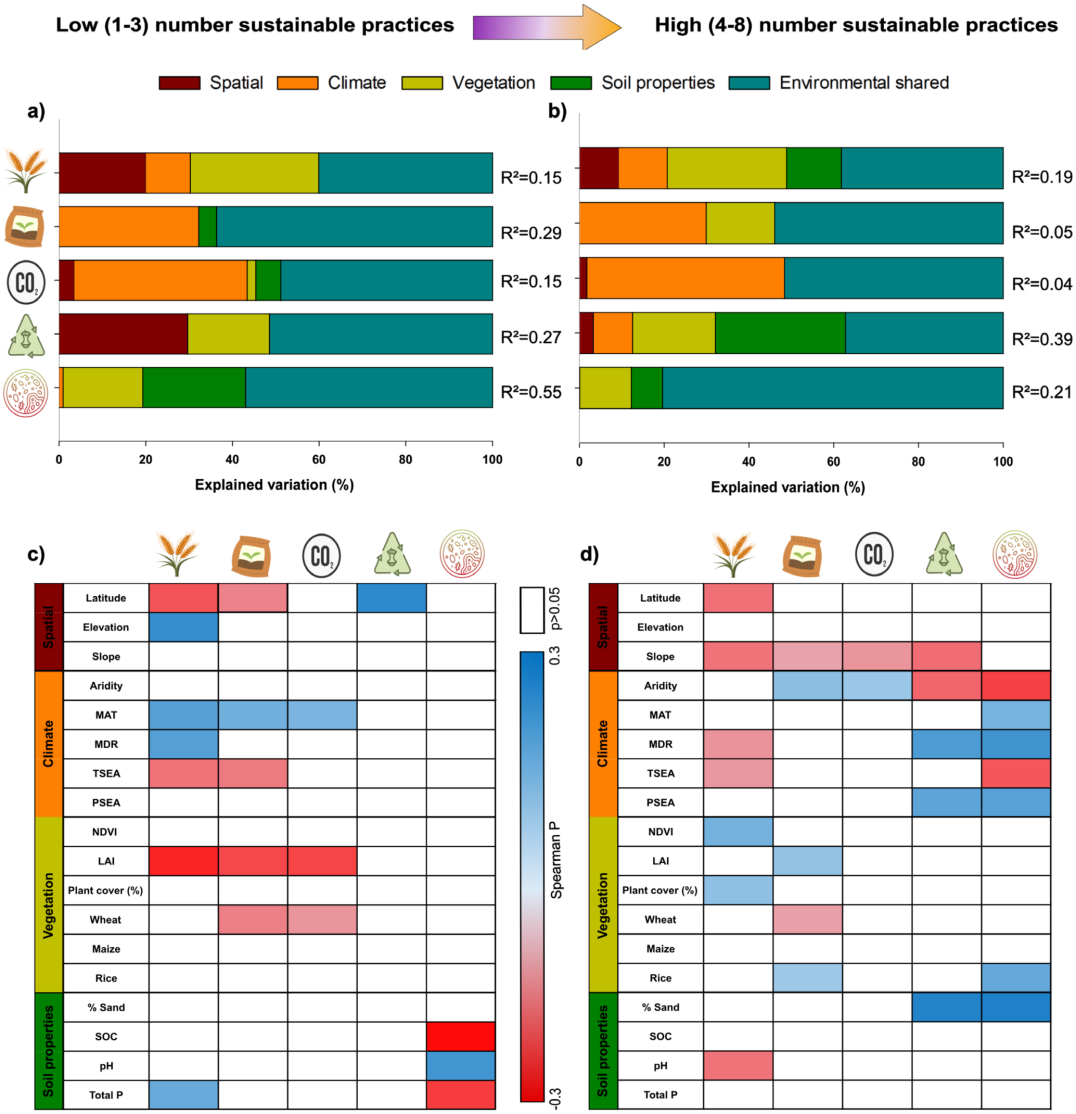
Environmental factors shape the effects (lnRR) of sustainable versus intensive managements on multiple ecosystem services under a relatively low (1–3) and high (4–8) number of management practices. Panels a and b show the relative importance of spatial, climate, vegetation and soil properties variables in modulating the effects (lnRR) of sustainability versus intensive management on crop yield, soil fertility, carbon sequestration, SOM decomposition and soil habitat under low (a; 1–3 practices) and high (b; 4–8 practices) number of sustainable practices. Environmental shared refers to the percent of shared variation in ecosystem services explained by all environmental variables. *R* ^2^ values express total variances corresponding to model adj. Panels c and d show the significant (*p* < 0.05) Spearman correlations among environmental factors and the ecosystem services: crop yield, soil fertility, carbon sequestration, SOM decomposition and soil habitat under low (c) and high (d) number of sustainable practices. The aridity index is positively correlated with water availability.

To assess how environmental context modulates the effects of sustainable management on agroecosystem services, we applied variation partitioning models (Figure 3). Our analyses showed that, both for low and high numbers of practices, climate is critical in shaping the effects of sustainable management on soil fertility and carbon sequestration, while vegetation properties such as productivity or type of crop are key to explaining the effect of sustainable management on crop yield, and soil habitat and SOM decomposition (Figure 3). Correlation analyses highlighted that environmental factors modulate how multiple sustainable practices affect agroecosystem services. We found that a high number of practices is particularly beneficial in sandy soils and regions with high precipitation seasonality (Figure 3). However, increases in aridity index revealed trade‐offs: while more practices enhanced soil carbon and fertility, they reduced benefits for habitat and OM decomposition. These trends were confirmed in stratified analyses by aridity (Figure S5). Additionally, multiple practices improved soil fertility in rice systems and mitigated potential nutrient limitations in wheat fields. Redundancy analyses showed low to moderate explanatory power across variable groups (Table S4). Finally, our analysis shows that practices such as crop rotation, limited tillage, and the incorporation of crop residues have overall positive effects on all agroecosystem services, including crop yield and soil habitat (Figure S4). When implemented together, these practices further enhanced the provision of all measured agroecosystem services (Figure S5).

### 3.3 North America, Eastern Europe and China Are Especially Dependent on a High Number of Sustainable Practices to Boost Ecoservices

**FIGURE 4:**
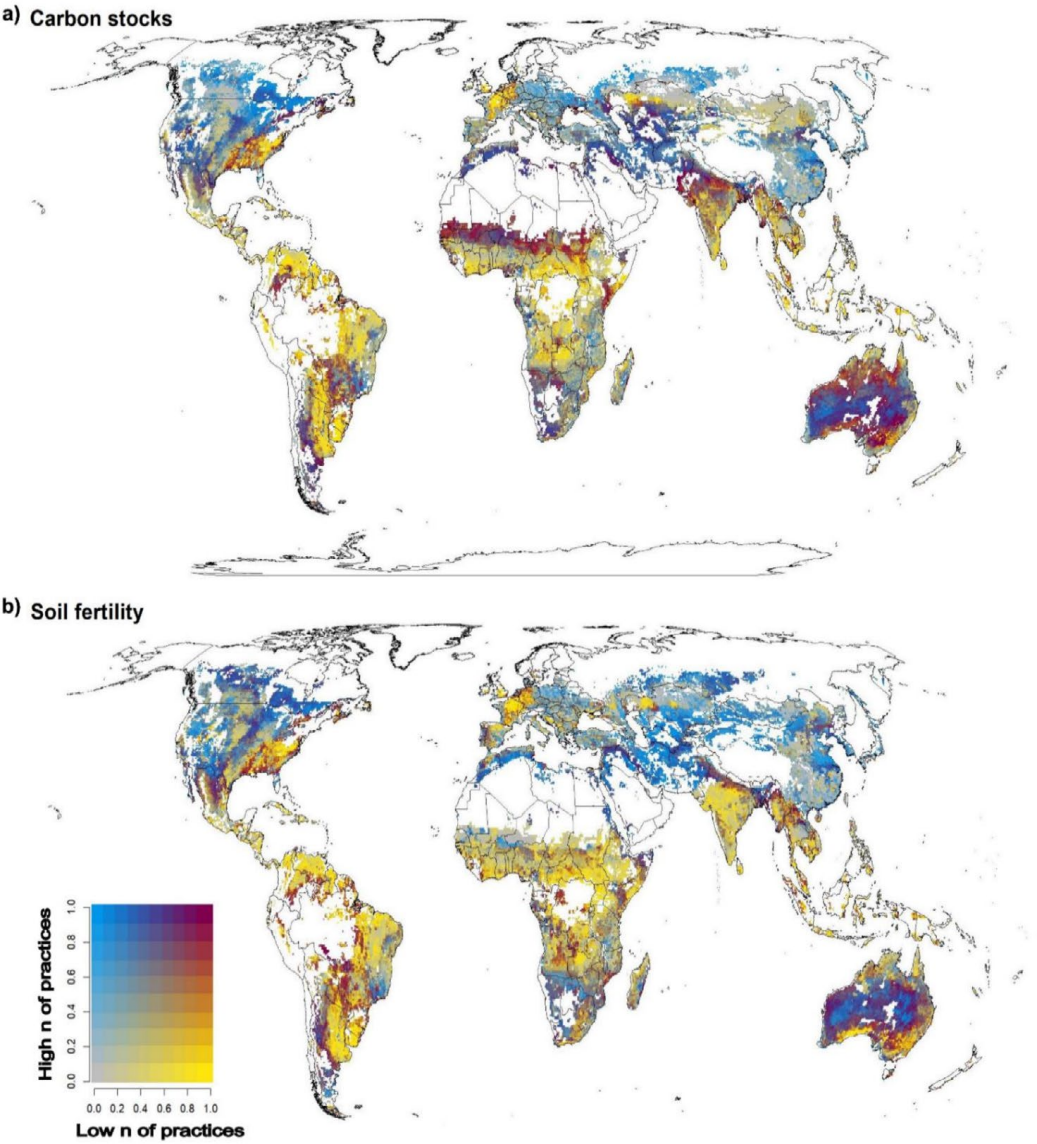
LnRR effects of sustainable versus intensive management practices on (a) soil carbon stocks and (b) fertility under a low (1–3 practices) and high (4–8 practices) number of management practices. Soil carbon stocks prediction power under low number and under high number of practices was *R* ^2^ = 0.48 and *R* ^2^ = 0.87, respectively. Soil fertility prediction power under low and high number of practices was *R* ^2^ = 0.58 and *R* ^2^ = 0.68, respectively. Maps are conducted at 25 km resolution. Blue indicates locations more dependent on a high number of sustainable management practices to support the ecosystem service, yellow indicates locations more dependent on a low number of sustainable management practices to support the ecosystem service and maroon indicates locations where low and high number of practices have more equivalent effects on ecosystem service. Locations with high uncertainty and areas not represented by environmental conditions in our study are shown in white.

We modelled the global distribution of the effects of multiple sustainable practices on soil carbon sequestration and fertility, two key services strongly supported by high numbers of practices (Figure 2) and critical for long‐term crop sustainability, food security, and climate regulation (Page et al. 2020). Using a random forest model, we generated global maps showing the effects of low and high numbers of sustainable practices on these services (Figure 4). The results reveal regions where either low or high practice intensity is crucial, as well as areas where both levels yield similar benefits. Regions with high environmental dissimilarity, as indicated by the Mahalanobis index, were excluded to avoid extrapolating beyond our dataset.

Our maps illustrate that, while the soil fertility and carbon sequestration of cereal fields in tropical regions seem to be more responsive to a low number of sustainable practices, temperate and cold regions are especially dependent on a high number of sustainable practices to boost these important agroecosystem services. This is in line with our correlation analyses showing that there is a greater positive effect of sustainable versus intensive management on soil fertility and carbon sequestration in warmer regions under a low number of sustainable practices (Figure 3). Overall, our map identifies China, Eastern Europe and North America as regions of the planet in which boosting soil fertility and carbon sequestration in cereal fields may largely depend on implementing a high number of sustainable practices. On the contrary, our analyses suggest that in tropical Africa, Southern India, and South America a low number of sustainable practices may already help support soil fertility and carbon sequestration.

## 4 Discussion

Understanding how to best apply sustainable farming is key to improving soil health, food security, and long‐term resilience amid global change. Our global analysis shows that using more sustainable practices boosts important agroecosystem services like crop yield, soil fertility, carbon storage and biodiversity. Practices such as crop rotation, reduced tillage and crop residue use are especially important. We also found that some regions rely more heavily on multiple practices to support soil carbon and fertility. Contrary to some current notions (e.g., Palm et al. 2014; FAO (Food and Agriculture Organization of the United Nations) 2011), our results suggest that implementing several sustainable practices together is essential for balancing food production and environmental health in the future.

### 4.1 Multiple Management Practices Are Needed to Support Sustainable Agriculture

The positive relationship between the number of sustainable practices and ecosystem services suggests potential complementarity among multiple sustainable practices that enhance nutrient cycles and soil functions. The significant improvement in soil carbon stocks with three or more practices highlights the role of sustainable agriculture in climate adaptation, by improving soil health and resilience (Liniger 2011). A clear fertility threshold emerged from six practices onward, suggesting cumulative and synergistic effects. Importantly, increasing sustainable practices showed neutral to positive impacts on yield, indicating that sustainability gains need not compromise production.

Sustainable practices increased the abundance of soil organisms, key drivers of fertility, decomposition, and carbon sequestration, enhancing microbial habitat even with a relatively small number of sustainable practices. Our results align with the idea that implementing more sustainable practices reduces agrochemical use and improves soil structure (Schmidt et al. 2011), creating a more stable environment that enhances water and nutrient retention, supports microbial diversity, and boosts soil sustainability and function (Hartmann et al. 2015; Lal 2015). The sensitivity of soil microbial communities to environmental changes is well established (Burman and Bengtsson‐Palme 2021; De Vries and Shade 2013), and thus the implementation of sustainable practices that buffer those changes may provide important long‐term benefits. We also found a positive effect of an increasing number of sustainable managements on soil biodiversity (Figure S2). Yet, the limited number of studies on soil biodiversity underscores the need for further research to better predict its patterns and clarify the effects of sustainable practices on soil health. Further research on the relationships between sustainable agricultural practices and soil biodiversity is crucial to developing comprehensive, sustainable farming systems. Overall, our findings suggest that the implementation of a large number of sustainable practices together results in a greater positive effect, thus improving soil structure and increasing capacity to retain nutrients, water and carbon (Sainju et al. 2003). This ‘strength in numbers’ approach, implementing multiple sustainable practices, promotes greater carbon sequestration in upper soil layers and boosts microbial activity, particularly of bacteria and mycorrhizal fungi, which are key to vital soil processes (Six et al. 2006). While our results underscore the benefits of increasing the number of sustainable practices, a key limitation is the lack of explicit evaluation of interactions among specific practices. It remains unclear whether observed effects are additive, synergistic or antagonistic. Future research should directly assess these interactions to refine management strategies and better understand the underlying mechanisms.

### 4.2 Environmental Context Fine‐Tunes the Effects of Multiple Sustainable Practices on Agroecosystem Services

The strong influence of climate and vegetation types on the influence of multiple sustainable practices on ecosystem services further highlights the need to tailor sustainable practices to local conditions. For instance, our results revealed an especially enhanced positive influence of multiple sustainable practices in sandy soils and high rainfall seasonality regions suggesting that these contexts may be more responsive to sustainable management practices. These findings underscore the importance of environmental context in modulating the benefits of sustainable management.

The observed trade‐offs in arid environments, where sustainable practices improved soil carbon and fertility but reduced habitat quality and decomposition, illustrate the complexity of agroecosystem responses and the necessity of context‐specific decision‐making (Briske 2017; Schröter et al. 2019). These findings advocate for more refined assessments, especially in heterogeneous landscapes where single management strategies may not deliver uniformly positive outcomes. Moreover, the effectiveness of crop rotation, limited tillage and residue incorporation as core practices suggests a practical starting point for many farming systems. Their synergistic effects not only benefit ecosystem function but also provide economic justification for adoption (Akinyi et al. 2021; Abubakar and Attanda 2013). By combining these complementary approaches, farmers can achieve both agronomic goals and ecological resilience, supporting a holistic transition toward sustainable agriculture. Taken together, our results demonstrate that maximizing the benefits of sustainable management requires consideration of multiple interacting factors. Strategies that are effective in one context may not be optimal in another, reinforcing the need for locally adapted solutions and further research into context‐dependency in agroecological systems. While the explanatory power of individual models was modest (*R* ^2^ values from 0.0009 to 0.1366), this is expected given the heterogeneity of global‐scale datasets. Local management practices, socio‐economic factors and data resolution likely contribute to unexplained variance and should be considered in future region‐specific analyses.

### 4.3 North America, Eastern Europe and China Are Especially Dependent on a High Number of Sustainable Practices to Boost Ecoservices

Global‐scale geographic patterns emphasise the context‐dependency of sustainable management outcomes at a global scale. The observed higher effectiveness of low‐input strategies in tropical regions may reflect greater baseline soil fertility, shorter histories of intensive land use, or greater inherent ecosystem resilience (Moura et al. 2016). In contrast, temperate regions like North America, Eastern Europe, and parts of China likely exhibit depleted baseline soil conditions due to prolonged intensive agriculture (European Environment Agency 2024; Joint Research Centre (JRC) 2024). This historical legacy has led to depleted soil conditions in many areas, making it necessary to implement multiple sustainable practices simultaneously to meaningfully restore soil health and ecosystem function. These patterns should also be interpreted in light of regional data availability. For instance, due to the limited number of studies for Eastern Europe (*n* = 4), the results might not fully capture the regional variability or broader trends. Our findings are consistent with previous correlation analyses (Figure 3) showing that temperature and climatic conditions modulate the response of soil fertility and carbon storage to sustainable practices. This further reinforces the importance of tailoring agricultural policy and management strategies to regional environmental conditions and historical land use.

The global maps generated in this study offer a practical tool for decision‐makers, enabling the identification of areas requiring significant investment in sustainable practices versus those where less intensive approaches may be sufficient. This intelligence can guide spatially targeted interventions, optimise resource allocation and inform agricultural development plans to maximise ecosystem service delivery while minimising expenditure. In conclusion, this analysis underscores that while a universally applicable strategy for sustainable agriculture is absent, regional variations contingent upon climate, soil characteristics and historical land utilisation are imperative for the formulation of efficacious and scalable solutions.

## 5 Conclusions

In summary, our work emphasizes the necessity of implementing multiple sustainable agricultural practices to enhance ecosystem services crucial for food security and environmental sustainability. By focusing on the number of practices in our analysis we de‐emphasize the actual nature and mode of action of the practices themselves, allowing us to make more generalizable statements without the need to understand the precise nature of synergistic interactions involving these practices. From a more practical standpoint, this method facilitates a rather quick and coarse‐level appraisal of the effort to achieve sustainability for a given field. Adopting comprehensive sustainable practices can generate synergistic benefits improving both economic viability and ecological health. This integrated strategy can help farmers maximize the potential of sustainable agriculture for positive business and environmental outcomes. However, real‐world implementation faces challenges. Socioeconomic factors like resource availability, labor, knowledge access, and policies greatly affect the adoption of integrated sustainable practices. While combining practices can yield additive or synergistic benefits, strategies must be tailored locally, supported by policies that enable context‐specific transitions. Sustainable agriculture requires technical solutions and inclusive systems that empower farmers to overcome barriers and unlock agroecosystem potential.

Our global dataset shows that the benefits of applying few or many sustainable practices vary by region, depending on local conditions such as climate, soil type, and farming systems. This highlights the importance of tailoring management strategies to regional contexts to improve soil quality and productivity. Sustainable practices also support long‐term soil fertility and ecosystem stability, crucial under climate change and population pressures. Our findings emphasize the value of synergistic practices for promoting both food security and ecosystem resilience, and point to the need for further research on how these practices interact across environmental gradients to encourage the global transition toward sustainable agriculture.

## Author Contributions

**Manuel Delgado‐Baquerizo and Luna Medrano:** conceptualization. **Manuel Delgado‐Baquerizo**, **Luna Medrano** and **Matthias C. Rilling:** methodology. **Manuel Delgado‐Baquerizo**, **Luna Medrano**, **Tadeo Sáez‐Sandino**, **Guiyao Zhou**, **Dongxue Tao**, **Kaiyan Zhai**, **Yue Yin** and **Tao Zhou:** investigation. **Luna Medrano** and **Tadeo Sáez‐Sandino:** visualisation. **Javier Alejandre**, **Gema del Río**, **Manuel Delgado‐Baquerizo**, **Margarita Ros**, **Jose Antonio Pascual**, **Antonio Rafael Sánchez‐Rodríguez**, **Raúl Ochoa‐Hueso**, **María del Mar Alguacil** and **Daniel Sacristán:** funding acquisition. **Manuel Delgado‐Baquerizo**, **Luna Medrano** and **Margarita Ros:** writing – original draft. **Luna Medrano**, **Margarita Ros**, **Tadeo Sáez‐Sandino**, **Guiyao Zhou**, **Dongxue Tao**, **Kaiyan Zhai**, **Yue Yin**, **Tao Zhou**, **Daniel Revillini**, **Jose Antonio Pascual**, **Antonio Rafael Sánchez‐Rodríguez**, **Raúl Ochoa‐Hueso**, **María del Mar Alguacil**, **Daniel Revillini**, **Javier Alejandre**, **Gema del Río**, **Matthias C. Rilling** and **Manuel Delgado‐Baquerizo:** writing – review and editing.

## Conflicts of Interest

The authors declare no conflicts of interest.

## Supporting information


**Data S1:** ele70276‐sup‐0001‐Supinfo.docx.

## Data Availability

Data was collected from the publications in Appendix S1. The repository contains all data, R scripts and Excel files necessary to reproduce the analyses and figures presented in the manuscript is available in this link: https://doi.org/10.6084/m9.figshare.30103849.v3. Identifier: 10.6084/m9.figshare.30103849.
