# Supplementary material for: Multiple Sustainable Practices Are Crucial for Enhancing the Provisioning of Agroecosystem Services Worldwide
Source: Ecol Lett. 2025 Dec 1;28(12):e70276. doi: 10.1111/ele.70276 (PMC12668573; doi:10.1111/ele.70276)
Supplement: Supplementary file 1 — Data S1: ele70276‐sup‐0001‐Supinfo.docx. [file ELE-28-0-s001.docx]

Supporting Information for

**Multiple sustainable practices are crucial for enhancing the provisioning of agroecosystem services worldwide**

Luna Medrano*^1^, Margarita Ros^2^, Tadeo Sáez-Sandino^3^, Guiyao Zhou^1^, Dongxue Tao^1,4^, Kaiyan Zhai^1^, Yue Yin^5^, Tao Zhou^1,6^, Dan Revillini^7^, Jose Antonio Pascual^2^, Antonio Rafael Sánchez-Rodríguez^8^, Raúl Ochoa-Hueso^7^, María del Mar Alguacil^2^, Daniel Sacristán^9^, Javier Alejandre^10^, Gema del Río^10^, Matthias C. Rilling^11-12^, Manuel Delgado-Baquerizo*^1^

^1^Laboratorio de Biodiversidad y Funcionamiento Ecosistémico. Instituto de Recursos Naturales y Agrobiología de Sevilla (IRNAS), Consejo Superior de Investigaciones Científicas (CSIC), Sevilla, Spain.

^2^Centro de Edafología y Biología Aplicada del Segura (CEBAS), CSIC, Campus Universitario de Espinardo, Murcia, Spain.

^3^Hawkesbury Institute for the Environment, Western Sydney University, Penrith, NSW, Australia.

^4^Institute of Grassland Science, Key Laboratory of Vegetation Ecology of the Ministry of Education, Jilin Songnen Grassland Ecosystem National Observation and Research Station, Northeast Normal University, Changchun, China

^5^State Key Laboratory of Urban and Regional Ecology, Research Center for Eco-Environmental Sciences, Chinese Academy of Sciences, Beijing, China

^6^School of Ecology and Northeast Asia Biodiversity Research Center, Northeast Forestry University, Harbin, Heilongjiang 150000, China

^7^Departamento de Biología, Área de Botánica. IVAGRO. Universidad de Cádiz, Puerto Real, Spain

^8^Unidad de Edafología, Departamento de Agronomía, Campus Universitario de Rabanales, Universidad de Córdoba, Córdoba, Spain

^9^Departamento de Biología Vegetal, Universidad de Valencia, Valencia, Spain

^10^Gabinete Técnico, Unión de Pequeños Agricultores y Ganaderos, Madrid, Spain

^11^Institute of Biology, Freie Universität Berlin, Berlin, Germany

^12^Berlin-Brandenburg Institute of Advanced Biodiversity Research, Berlin, Germany

*Corresponding authors.

**Email:** Luna Medrano (luna.m.g.27@csic.es); Manuel Delgado-Baquerizo (M.delgado.baquerizo@csic.es)

This PDF file includes:

Supplementary Figs 1 to 4

Supplementary Tables 1 to 3

Appendix S1


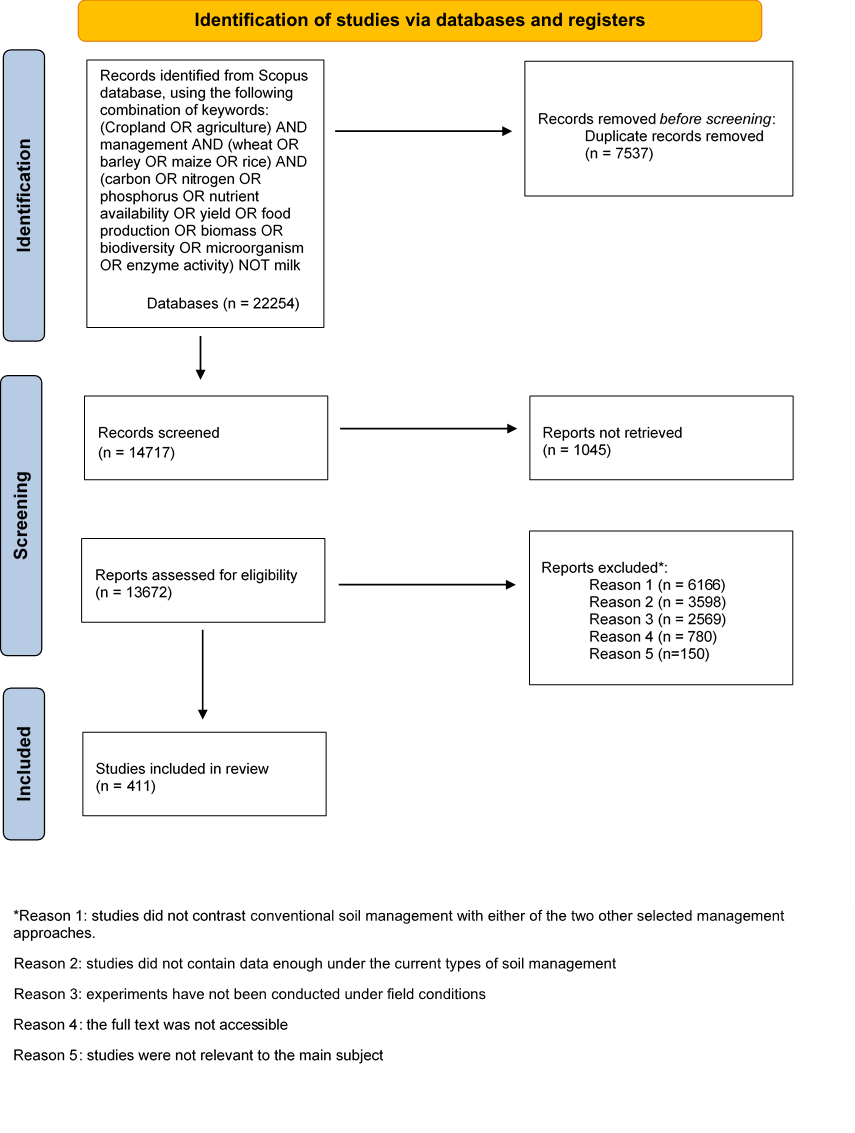


Reason 1: studies did not contrast intensive agriculture management with at least one of the selected sustainable agriculture practices.

Reason 2: studies did not contain data enough under the current types of soil management

Reason 3: experiments were not conducted under field conditions

Reason 4: the full text was not accessible

Reason 5: studies were not relevant to the main subject

**Supplementary Figure 1 | PRISMA flow diagram including the information association with our systematic review of the literature.** See Appendix S1 below for a complete list of selected publications.

**Supplementary Figure 2 | Effect of an increasing number of sustainable agriculture practices (vs. intensive agriculture) on soil biodiversity (i.e., lnRR).** The error bars show 95% confidence intervals (CI), color dots indicate significant difference at *p*-value < 0.05, significantly positive in red and significantly negative in blue. Non-significant changes are denoted by gray dots.


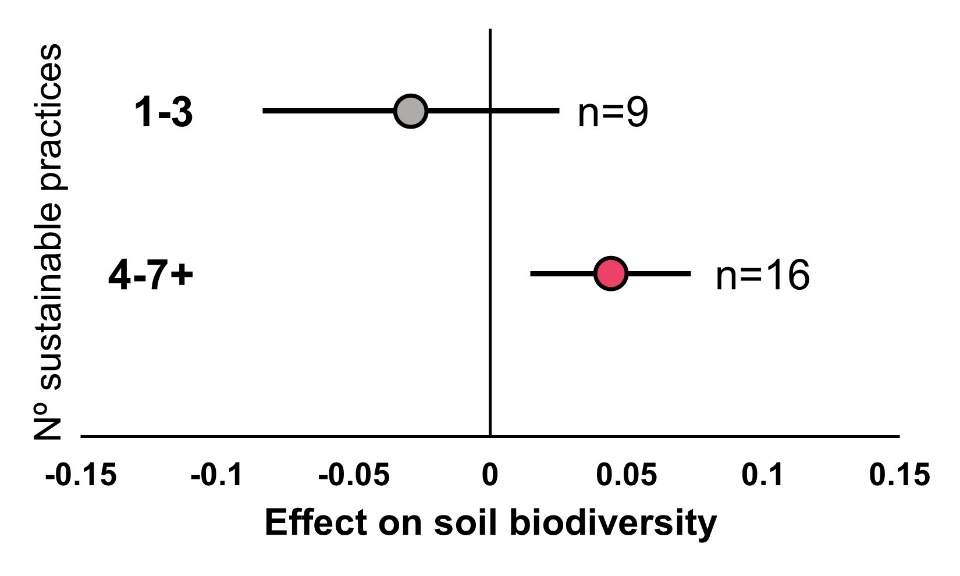


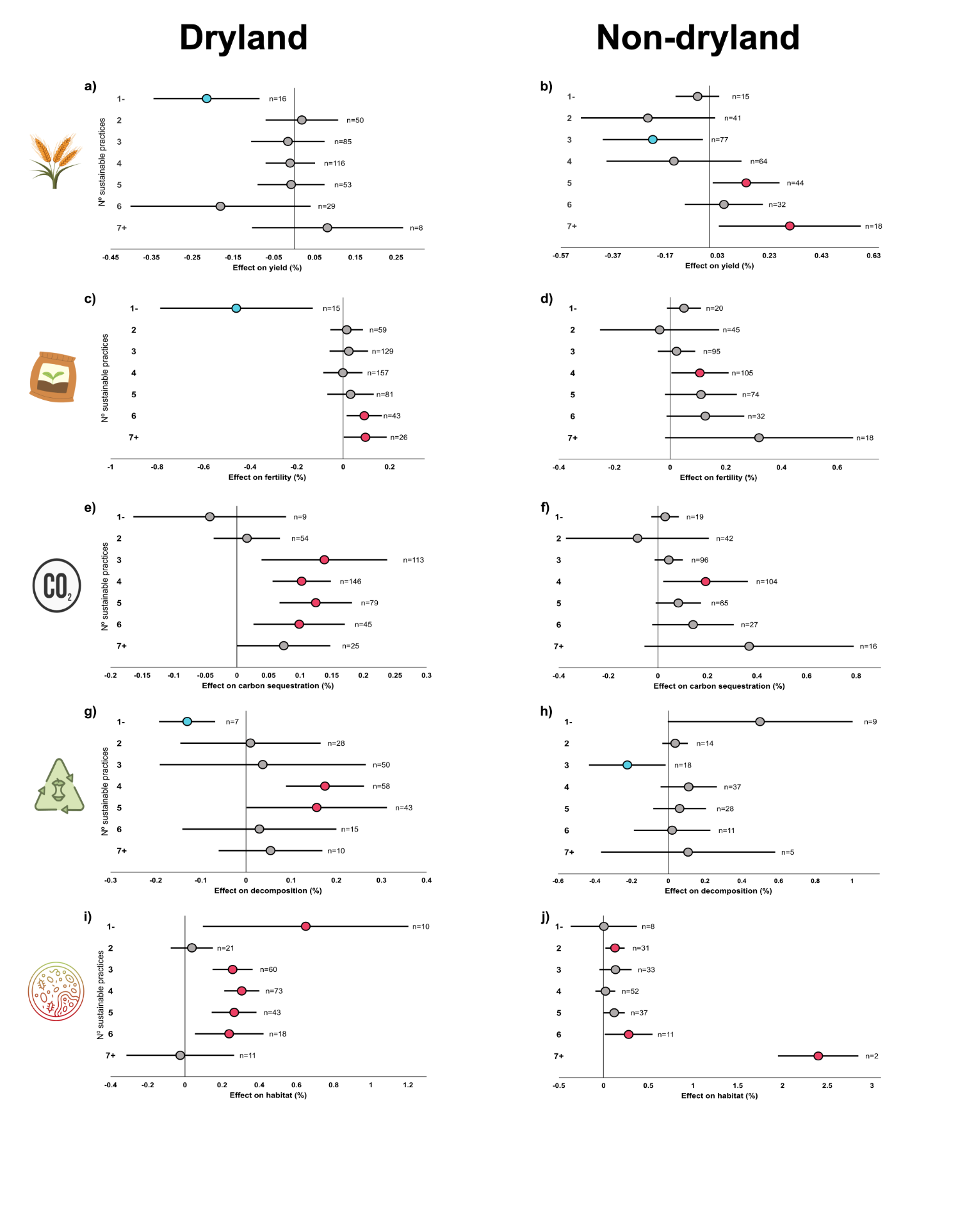


**Supplementary Figure 3 | Effect (lnRR) of an increasing number of sustainable agriculture versus intensive agriculture on multiple ecosystem services: a** crop yield; **b** soil fertility; **c** carbon sequestration; **d** SOM decomposition and, **d** soil habitat, across contrasting aridity levels. The error bars show 95% confidence intervals (CI), colored points indicate significant difference at *p*-value < 0.05, significantly positive in red and significantly negative in blue. Non-significant changes are denoted by gray points. Numbers (n) indicate the number of studies. Drylands = Aridity Index < 0.65; Non-drylands = Aridity Index > 0.65.


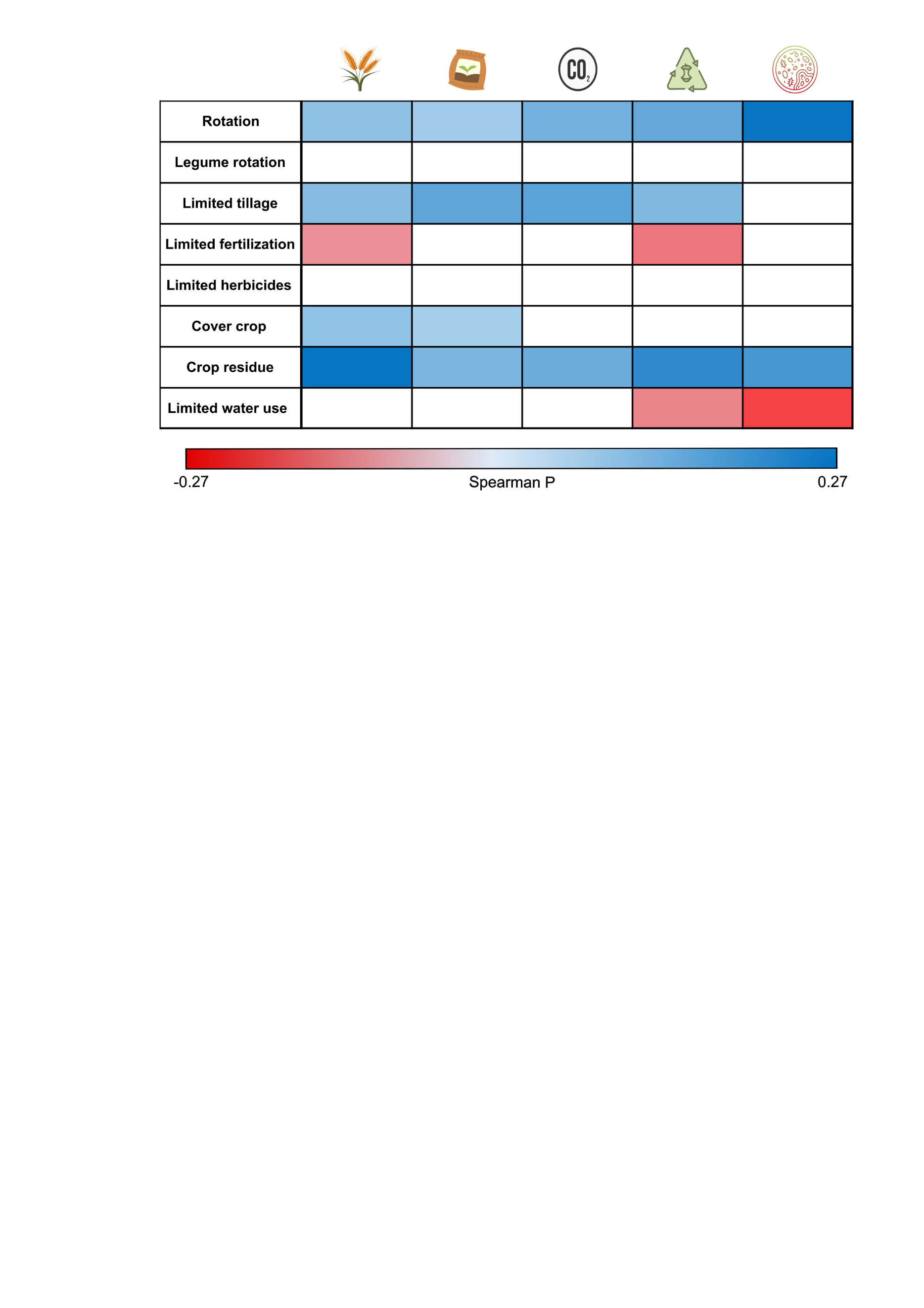


**Supplementary Figure 4 | Spearman correlations** between individual sustainable agricultural practices and the responses (lnRR) of ecosystem services (crop yield, soil fertility, carbon sequestration, SOM decomposition and soil habitat) to different groups of sustainable managements. Only significant correlations (P < 0.05) are included in this figure. Non-significant correlations are included in white color.


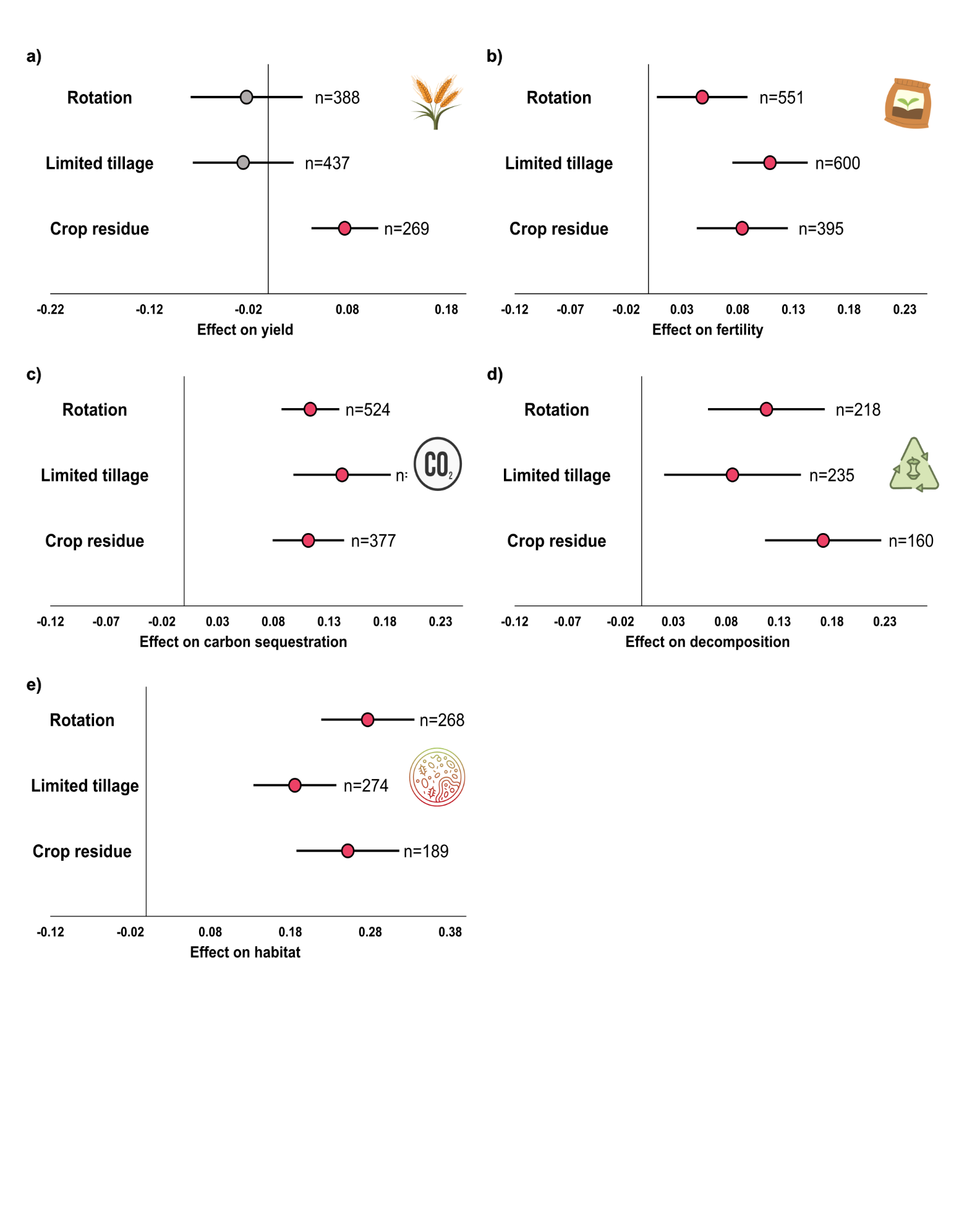


**Supplementary Figure 5 | Effect of rotation, limited tillage and crop residue (vs intensive practices such as no rotation, conventional tillage and crop residue removal, respectively on**: **a** crop yield; **b** soil fertility; **c** carbon sequestration; **d** SOM decomposition and, **d** soil habitat. The error bars show 95% confidence intervals (CI), colored dots indicate significant difference at *p*-value < 0.05, with significantly positive in red and significantly negative in blue. Non-significant changes are denoted by gray dots.

| **Ecosystem services** | | **Ecosystem attribute**  **Supplementary Table 1 \|** Individual ecosystem attributes that composed ecosystem service response variables in our analyses. |  |
| --- | --- | --- | --- |
| Yield | Total biomass, aboveground biomass, belowground biomass, grain yield | | |
| Soil fertility | Soil total nitrogen (N) and phosphorus (P); Soil inorganic N, P; Soil organic N, P | | |
| Carbon sequestration | Soil organic matter (SOM); mineral, organic, available, labile, dissolved organic carbon, carbon sequestration rates | | |
| SOM decomposition | Potential N, C mineralization; enzymatic activities: rubisco, protease, NAG, β-glucosidase, urease, phosphatase, FDA, DHA, sucrase, cellulase, amylase, catalase, peroxidase; β-respiration, microbial metabolic quotient (${qCO}_{2}$), metabolic quotient, microbial quotient | | |
| Soil habitat | Bacterial abundance and biomass; fungal abundance, population and biomass; microarthropod and nematode abundance and biomass, earthworm density and biomass | | |
| Soil biodiversity | Shannon index for bacteria, fungi, earthworms, microarthropods and nematodes | | |

| **Management practice** | **Category** | **Classification** | **Rationale** |
| --- | --- | --- | --- |
| Rotation | No rotation | 0 | Crop rotation offers potential benefits to farmers and environment (Zou et al. 2024) |
|  | Rotation | 1 |  |
| Type of rotation | No legume rotation | 0 | Legumes can increase nitrogen inputs reducing the need for synthetic fertilizers (Diacono et al. 2019) |
|  | Legume rotation | 1 |  |
| Tillage | Conventional tillage | 0 | Reducing tillage practices promotes land conservation and reduces soil impacts (Wang et al. 2024) |
|  | Reduced tillage | 1 |  |
|  | No tillage | 1 |  |
| Fertilization | Synthetic chemical fertilization | 0 | Organic fertilization sustains the soil and environments in the long term, reduces the input cost and contributes to recycling of waste materials (Verma et al. 2020) |
|  | Reduced fertilization | 0 |  |
|  | No fertilization | 1 |  |
|  | Animal manure | 1 |  |
|  | Organic soil amendment | 1 |  |
|  | Compost | 1 |  |
| Plant protection | Herbicides | 0 | Synthetic weed management amendments contaminate and can degrade the environment, biodiversity and food safety; biocontrol activity benefits plant growth and environmental sustainability (Parven et al. 2025) |
|  | Fungicides | 0 |  |
|  | Pesticides | 0 |  |
|  | Insecticides | 0 |  |
|  | Plastic mulch | 0 |  |
|  | Mechanical | 1 |  |
|  | Bio-control | 1 |  |
|  | No protection | 1 |  |
| Cover crop | No cover | 0 | Cover crops regulate nutrient cycling, soil fertility, meteorological events, and benefit soil and plant biodiversity (Quintarelli et al. 2022) |
|  | Green manuring | 1 |  |
|  | Permanent cover | 1 |  |
| Crop residue | Residue removed | 0 | Wasting and burning crop residues results in heavy loss of soil nutrients and organic carbon, incorporating or retaining residues in to the soil promotes soil health, increasing habitat and soil nutrients (Maurya et al. 2020) |
|  | Crop residue burned | 0 |  |
|  | Crop residue incorporated | 1 |  |
|  | Crop residue retained | 1 |  |
| Water use | Water irrigation | 0 | The efficiency of irrigation is conventionally very low and over-watering is common. Improvements in irrigation application, water saving and reuse of treated wastewater increase water efficiency and promote environmental protection (Chartzoulakis & Bertaki 2015) |
|  | Flooding irrigation | 0 |  |
|  | Non-irrigated | 1 |  |
|  | Sewage irrigation | 1 |  |
|  | Controlled/reduced irrigation | 1 |  |
|  | Superficial/drip irrigation | 1 |  |

**Supplementary Table 2 |** Classification of the different treatments and agricultural practices into intensive agricultural practices (0) and sustainable agricultural practices (1). See rationale for how these categories were designed.

**Supplementary Table 3 | R**esponse ratio (LnRR) and 95% confidence intervals (CI 95%) of sustainable management effects on each ecosystem service, separated by the number of implemented sustainable agricultural practices (from 1 to ≥7). This table supports detailed inspection of the effect size trends described in the main text and complements Figure 2.

| Ecosystem service | Nº practices | LnRR | CI 95% |
| --- | --- | --- | --- |
| Yield | 1 | -0.1411 | 0.0884 |
|  | 2 | -0.0958 | 0.1259 |
|  | 3 | -0.1114 | 0.1028 |
|  | 4 | -0.0542 | 0.0989 |
|  | 5 | 0.0598 | 0.0736 |
|  | 6 | -0.0568 | 0.1308 |
|  | 7+ | 0.2384 | 0.1931 |
|  | 1 | -0.1879 | 0.1749 |
|  | 2 | -0.0079 | 0.0988 |
|  | 3 | 0.0236 | 0.0551 |
| Soil fertility | 4 | 0.0422 | 0.0650 |
|  | 5 | 0.0693 | 0.0801 |
|  | 6 | 0.1059 | 0.0720 |
|  | +7 | 0.1868 | 0.1444 |
|  | 1 | 0.0024 | 0.0575 |
|  | 2 | -0.0273 | 0.1271 |
|  | 3 | 0.0950 | 0.0597 |
| Carbon sequestration | 4 | 0.1398 | 0.0756 |
|  | 5 | 0.1060 | 0.0514 |
|  | 6 | 0.1147 | 0.0743 |
|  | +7 | 0.1888 | 0.1659 |
|  | 1 | 0.2460 | 0.3554 |
|  | 2 | 0.0187 | 0.1036 |
|  | 3 | -0.0319 | 0.1762 |
| SOM decomposition | 4 | 0.1500 | 0.0783 |
|  | 5 | 0.1191 | 0.1081 |
|  | 6 | 0.0257 | 0.1223 |
|  | +7 | 0.0720 | 0.1343 |
|  | 1 | 0.3620 | 0.3538 |
|  | 2 | 0.0916 | 0.0772 |
|  | 3 | 0.2126 | 0.0931 |
| Soil habitat | 4 | 0.1872 | 0.0756 |
|  | 5 | 0.1964 | 0.0850 |
|  | 6 | 0.2543 | 0.1428 |
|  | +7 | 0.3480 | 0.5995 |
| Soil biodiversity | 1-3 | -0.0289 | 0.0543 |
|  | 4-7+ | 0.0441 | 0.0294 |

| Ecosystem service | Variable group | Df | $\boldsymbol{R}^{\boldsymbol{2}}$(explained variance) | F value | p-value |  |
| --- | --- | --- | --- | --- | --- | --- |
|  | Spatial | 3 | 0.115 | 4.3823 | 0.005* |  |
| Yield (1-3) | Climate | 5 | 0.0083 | 1.8421 | 0.109 |  |
|  | Vegetation | 6 | 0.1366 | 2.5857 | 0.032* |  |
|  | Soil properties | 4 | 0.006544 | 1.7978 | 0.132 |  |
| Yield (4-8) | Spatial | 3 | 0.0023 | 0.8937 | 0.4 |  |
|  | Climate | 5 | 0.0126 | 3.0917 | 0.018* |  |
|  | Vegetation | 6 | 0.0162 | 3.3715 | 0.004** |  |
|  | Soil properties | 4 | 0.0136 | 4.211 | 0.004** |  |
|  | Spatial | 3 | 0.0099 | 2.6523 | 0.059 |  |
| Soil fertility (1-3) | Climate | 5 | 0.0345 | 6.0713 | 0.001* |  |
|  | Vegetation | 6 | 0.0051 | 0.668 | 0.665 |  |
|  | Soil properties | 4 | 0.0117 | 2.3584 | 0.051 |  |
|  | Spatial | 3 | 0.0009 | 0.4489 | 0.646 |  |
| Soil fertility (4-8) | Climate | 5 | 0.0096 | 3.0289 | 0.016* |  |
|  | Vegetation | 6 | 0.0044 | 1.1316 | 0.314 |  |
|  | Soil properties | 4 | 0.0045 | 1.753 | 0.141 |  |
|  | Spatial | 3 | 0.0117 | 1.9256 | 0.149 |  |
| Carbon sequestration (1-3) | Climate | 5 | 0.0395 | 4.1596 | 0.004* |  |
|  | Vegetation | 6 | 0.0139 | 1.1303 | 0.334 |  |
|  | Soil properties | 4 | 0.0166 | 2.0605 | 0.082 |  |
|  | Spatial | 3 | 0.0023 | 2.0323 | 0.116 |  |
| Carbon sequestration (4-8) | Climate | 5 | 0.0068 | 3.6391 | 0.003** |  |
|  | Vegetation | 6 | 0.0016 | 0.6877 | 0.642 |  |
|  | Soil properties | 4 | 0.0038 | 2.4853 | 0.049* |  |
|  | Spatial | 3 | 0.0231 | 4.1558 | 0.016* |  |
| SOM decomposition (1-3) | Climate | 5 | 0.0098 | 0.919 | 0.482 |  |
|  | Vegetation | 6 | 0.0180 | 1.4768 | 0.22 |  |
|  | Soil properties | 4 | 0.0113 | 1.3659 | 0.235 |  |
|  | Spatial | 3 | 0.0056 | 1.5306 | 0.205 |  |
| SOM decomposition (4-8) | Climate | 5 | 0.0166 | 2.9216 | 0.017* |  |
|  | Vegetation | 6 | 0.0229 | 3.5118 | 0.005** |  |
|  | Soil properties | 4 | 0.0067 | 1.3902 | 0.239 |  |
|  | Spatial | 3 | 0.0064 | 0.7495 | 0.488 |  |
| Soil habitat (1-3) | Climate | 5 | 0.0310 | 2.3595 | 0.039* |  |
|  | Vegetation | 6 | 0.0839 | 6.8394 | 0.001*** |  |
|  | Soil properties | 4 | 0.0460 | 4.7297 | 0.007** |  |
|  | Spatial | 3 | 0.0157 | 2.6084 | 0.07 |  |
| Soil habitat (4-8) | Climate | 5 | 0.0290 | 2.986 | 0.009** |  |
|  | Vegetation | 6 | 0.0107 | 0.8524 | 0.527 |  |
|  | Soil properties | 4 | 0.0232 | 2.9472 | 0.023* |  |

**Supplementary Table 4 |** Summary of redundancy analysis (RDA) models evaluating the effect of different groups of explanatory variables on ecosystem services. Each model tests the contribution of a group of variables: (1) spatial (latitude, elevation, slope), (2) climatic (aridity, MAT, MDR, TSEA, PSEA), (3) vegetation (NDVI, LAI, plant cover, crop type), and (4) soil properties (sand content, SOC, pH, total phosphorus). The table reports the degrees of freedom (Df), variance explained (R²), F-value, and p-value from permutation tests (999 permutations). Significant models are marked (*p < 0.05; **p < 0.01). These results provide additional context to the variation partitioning analysis shown in Figure 3, highlighting the individual explanatory power of environmental and edaphic factors.

**Supplementary Table 5 |** Variance Inflation Factor (VIF) index measuring the estimated coefficient of collinearity. All the variables with a VIF > 5 were removed from analyses.

| Low number of practices (0-3) | | High number of practices (4-8) | |
| --- | --- | --- | --- |
| Variable | **VIF** | **Variable** | **VIF** |
| Plant cover (%) | 3.232759 | NDVI | 4.039692 |
| pH | 3.207885 | Plant cover (%) | 3.366945 |
| Aridity index | 3.170882 | MAT | 3.156117 |
| TSEA | 2.976987 | TSEA | 2.93206 |
| MAT | 2.960045 | pH | 2.706847 |
| SOC | 2.759666 | Aridity index | 2.409056 |
| MDR | 2.441771 | MDR | 2.261626 |
| NDVI | 2.411682 | Elevation | 2.11696 |
| PSEA | 2.309123 | SOC | 2.04553 |
| LAI | 1.955534 | LAI | 1.902319 |
| Elevation | 1.831594 | PSEA | 1.693681 |
| Fine texture | 1.576748 | Fine texture | 1.506891 |
| Slope | 1.486065 | Slope | 1.390974 |
| P total | 1.202899 | P total | 1.073078 |

**Appendix S1 List of publications selected in our meta-analysis.** See Supplementary Figure 1 for details.

1. Abán, C. L. et al. Service crops improve a degraded monoculture system by changing common bean rhizospheric soil microbiota and reducing soil-borne fungal diseases. FEMS Microbiology Ecology 97, fiaa258 (2021).

2. Abbas, F. et al. Transforming a Valuable Bioresource to Biochar, Its Environmental Importance, and Potential Applications in Boosting Circular Bioeconomy While Promoting Sustainable Agriculture. Sustainability 13, 2599 (2021).

3. Abbasi, M. K. & Tahir, M. M. Economizing Nitrogen Fertilizer in Wheat through Combinations with Organic Manures in Kashmir, Pakistan. Agronomy Journal 104, 169–177 (2012).

4. Abbasi, M. K., Tahir, M. M. & Rahim, N. Effect of N fertilizer source and timing on yield and N use efficiency of rainfed maize (Zea mays L.) in Kashmir–Pakistan. Geoderma 195–196, 87–93 (2013).

5. Abrar, M. M. et al. Long‐term manure application enhances organic carbon and nitrogen stocks in Mollisol subsoil. Land Degrad Dev 34, 815–832 (2023).

6. Abril, A. Labile and Recalcitrant Carbon in Crop Residue and Soil under No-Till Practices in Central Region of Argentina. TOASJ 7, 32–39 (2013).

7. Adekiya, A. O., Ogunboye, O. I., Ewulo, B. S. & Olayanju, A. Effects of Different Rates of Poultry Manure and Split Applications of Urea Fertilizer on Soil Chemical Properties, Growth, and Yield of Maize. The Scientific World Journal 2020, 4610515 (2020).

8. Agele, S. O., Ewulo, B. S. & Oyewusi, I. K. Effects of Some Soil Management Systems on Soil Physical Properties, Microbial Biomass and Nutrient Distribution Under Rainfed Maize Production in a Humid Rainforest Alfisol. Nutr Cycl Agroecosyst 72, 121–134 (2005).

9. Akhtar, K. et al. Integrated use of straw mulch with nitrogen fertilizer improves soil functionality and soybean production. Environment International 132, 105092 (2019).

10. Akmal, M., Shah, A. & Ali, J. Growth, radiation use efficiency and grain yield of wheat as influenced by nitrogen, tillage, and crop residue management. Journal of Plant Nutrition 41, 2032–2047 (2018).

11. Akoto-Danso, E. K. et al. Agronomic effects of biochar and wastewater irrigation in urban crop production of Tamale, northern Ghana. Nutr Cycl Agroecosyst 115, 231–247 (2019).

12. Al Aasmi, A. et al. Impacts of Slow-Release Nitrogen Fertilizer Rates on the Morpho-Physiological Traits, Yield, and Nitrogen Use Efficiency of Rice under Different Water Regimes. Agriculture 12, 86 (2022).

13. Alarefee, H., Ishak, C. F., Karam, D. S. & Othman, R. Efficiency of Rice Husk Biochar with Poultry Litter Co-Composts in Oxisols for Improving Soil Physico-Chemical Properties and Enhancing Maize Performance. Agronomy 11, 2409 (2021).

14. Alarefee, H., Ishak, C. F., Karam, D. S. & Othman, R. Efficiency of Rice Husk Biochar with Poultry Litter Co-Composts in Oxisols for Improving Soil Physico-Chemical Properties and Enhancing Maize Performance. Agronomy 11, 2409 (2021).

15. Alavaisha, E., Manzoni, S. & Lindborg, R. Different agricultural practices affect soil carbon, nitrogen and phosphorous in Kilombero -Tanzania. Journal of Environmental Management 234, 159–166 (2019).

16. Alhassan, A.-R. M., Yang, C., Ma, W. & Li, G. Influence of conservation tillage on Greenhouse gas fluxes and crop productivity in spring-wheat agroecosystems on the Loess Plateau of China. PeerJ 9, e11064 (2021).

17. Alijani, K., Bahrani, M. J. & Kazemeini, S. A. Short-term responses of soil and wheat yield to tillage, corn residue management and nitrogen fertilization. Soil and Tillage Research 124, 78–82 (2012).

18. Aller, D. et al. Biochar Age and Crop Rotation Impacts on Soil Quality. Soil Science Society of America Journal 81, 1157–1167 (2017).

19. Álvaro-Fuentes, J., Arrúe, J. L., Gracia, R. & López, M. V. Tillage and cropping intensification effects on soil aggregation: Temporal dynamics and controlling factors under semiarid conditions. Geoderma 145, 390–396 (2008).

20. Ambus, P. & Jensen, E. S. Crop Residue Management Strategies to Reduce N-Losses—Interaction with Crop N Supply. Communications in Soil Science and Plant Analysis 32, 981–996 (2001).

21. Anning, D. K. et al. Maize Straw Return and Nitrogen Rate Effects on Potato (Solanum tuberosum L.) Performance and Soil Physicochemical Characteristics in Northwest China. Sustainability 13, 5508 (2021).

22. Ansari, M. A. et al. Green manuring and crop residue management: Effect on soil organic carbon stock, aggregation, and system productivity in the foothills of Eastern Himalaya (India). Soil and Tillage Research 218, 105318 (2022).

23. Apesteguía, M. et al. Tillage Effects on Soil Quality after Three Years of Irrigation in Northern Spain. Sustainability 9, 1476 (2017).

24. Araya, T. et al. Medium-term effects of conservation agriculture based cropping systems for sustainable soil and water management and crop productivity in the Ethiopian highlands. Field Crops Research 132, 53–62 (2012).

25. Araya, T. et al. Influence of 9 years of permanent raised beds and contour furrowing on soil health in conservation agriculture based systems in Tigray region, Ethiopia. Land Degradation & Development 32, 1525–1539 (2021).

26. Arcand, M. M., Helgason, B. L. & Lemke, R. L. Microbial crop residue decomposition dynamics in organic and conventionally managed soils. Applied Soil Ecology 107, 347–359 (2016).

27. Ardenti, F., Capra, F., Lommi, M., Fiorini, A. & Tabaglio, V. Long-term C and N sequestration under no-till is governed by biomass production of cover crops rather than differences in grass vs. legume biomass quality. Soil and Tillage Research 228, 105630 (2023).

28. Asuming-Brempong, S. et al. Changes in the biodiversity of microbial populations in tropical soils under different fallow treatments. Soil Biology and Biochemistry 40, 2811–2818 (2008).

29. Aziz, I., Mahmood, T. & Islam, K. R. Effect of long term no-till and conventional tillage practices on soil quality. Soil and Tillage Research 131, 28–35 (2013).

30. Aziz, O. et al. Increasing water productivity, nitrogen economy, and grain yield of rice by water saving irrigation and fertilizer-N management. Environ Sci Pollut Res 25, 16601–16615 (2018).

31. Babu, S. et al. Impact of land configuration and organic nutrient management on productivity, quality and soil properties under baby corn in Eastern Himalayas. Sci Rep 10, 16129 (2020).

32. Badagliacca, G. et al. Long-term effects of contrasting tillage systems on soil C and N pools and on main microbial groups differ by crop sequence. Soil and Tillage Research 211, 104995 (2021).

33. Badagliacca, G. et al. Early Effects of No-Till Use on Durum Wheat (Triticum durum Desf.): Productivity and Soil Functioning Vary between Two Contrasting Mediterranean Soils. Agronomy 12, 3136 (2022).

34. Baghel, J. K. et al. Effect of conservation agriculture and weed management on weeds, soil microbial activity and wheat (Triticum aestivum) productivity under a rice (Oryza sativa)-wheat cropping system. Indian J Agri Sci 88, 1709–1716 (2018).

35. Bakht, J., Shafi, M., Jan, M. T. & Shah, Z. Influence of crop residue management, cropping system and N fertilizer on soil N and C dynamics and sustainable wheat (Triticum aestivum L.) production. Soil and Tillage Research 104, 233–240 (2009).

36. Baral, B. R. et al. Increasing nitrogen use efficiency in rice through fertilizer application method under rainfed drought conditions in Nepal. Nutr Cycl Agroecosyst 118, 103–114 (2020).

37. Baruah, A., Baruah, K. K. & Bhattacharyya, P. Comparative Effectiveness of Organic Substitution in Fertilizer Schedule: Impacts on Nitrous Oxide Emission, Photosynthesis, and Crop Productivity in a Tropical Summer Rice Paddy. Water Air Soil Pollut 227, 410 (2016).

38. Bauza-Kaszewska, J., Breza-Boruta, B., Lemańczyk, G. & Lamparski, R. Effects of Eco-Friendly Product Application and Sustainable Agricultural Management Practices on Soil Properties and Phytosanitary Condition of Winter Wheat Crops. Sustainability 14, 15754 (2022).

39. Benbi, D. K. et al. Differences in soil organic carbon pools and biological activity between organic and conventionally managed rice-wheat fields. Org. Agr. 8, 1–14 (2018).

40. Bhaduri, D., Purakayastha, T. J., Patra, A. K., Singh, M. & Wilson, B. R. Biological indicators of soil quality in a long-term rice–wheat system on the Indo-Gangetic plain: combined effect of tillage–water–nutrient management. Environ Earth Sci 76, 202 (2017).

41. Bhattacharyya, P. et al. Greenhouse gas emission in relation to labile soil C, N pools and functional microbial diversity as influenced by 39 years long-term fertilizer management in tropical rice. Soil and Tillage Research 129, 93–105 (2013).

42 Bhattacharyya, R. et al. Aggregate-associated N and global warming potential of conservation agriculture-based cropping of maize-wheat system in the north-western Indo-Gangetic Plains. Soil and Tillage Research 182, 66–77 (2018).

43. Bhattacharyya, R. et al. Conservation agriculture effects on soil organic carbon accumulation and crop productivity under a rice–wheat cropping system in the western Indo-Gangetic Plains. European Journal of Agronomy 70, 11–21 (2015).

44. Bi, L. et al. Long-term effects of organic amendments on the rice yields for double rice cropping systems in subtropical China. Agriculture, Ecosystems & Environment 129, 534–541 (2009).

45. Bi, Q.-F. et al. Partial replacement of inorganic phosphorus (P) by organic manure reshapes phosphate mobilizing bacterial community and promotes P bioavailability in a paddy soil. Science of The Total Environment 703, 134977 (2020).

46. Birkhofer, K. et al. Long-term organic farming fosters below and aboveground biota: Implications for soil quality, biological control and productivity. Soil Biology and Biochemistry 40, 2297–2308 (2008).

47. Bisht, J. K., Chandra, S., Singh, R. D. & Gupta, H. S. Role of N in yield enhancement of clipped wheat. (211). Tropical Agriculture (2004).

48. Biswakarma, N. et al. Five years integrated crop management in direct seeded rice–zero till wheat rotation of north-western India: Effects on soil carbon dynamics, crop yields, water productivity and economic profitability. Agriculture, Ecosystems & Environment 318, 107492 (2021).

49. Bosch-Serra, A. D., Yagüe, M. R., Valdez, A. S. & Domingo-Olivé, F. Dairy cattle slurry fertilization management in an intensive Mediterranean agricultural system to sustain soil quality while enhancing rapeseed nutritional value. Journal of Environmental Management 273, 111092 (2020).

50. Boudiar, R. et al. Influence of Tillage and Cropping Systems on Soil Properties and Crop Performance under Semi-Arid Conditions. Sustainability 14, 11651 (2022).

51. Bouraima, A.-K., He, B. & Tian, T. Runoff, nitrogen (N) and phosphorus (P) losses from purple slope cropland soil under rating fertilization in Three Gorges Region. Environ Sci Pollut Res 23, 4541–4550 (2016).

52. Brar, B. S., Singh, K., Dheri, G. S., & Balwinder-Kumar. Carbon sequestration and soil carbon pools in a rice?wheat cropping system: Effect of long-term use of inorganic fertilizers and organic manure. Soil and Tillage Research 128, 30–36 (2013).

53. Breitkreuz, C., Herzig, L., Buscot, F., Reitz, T. & Tarkka, M. Interactions between soil properties, agricultural management and cultivar type drive structural and functional adaptations of the wheat rhizosphere microbiome to drought. Environmental Microbiology 23, 5866–5882 (2021).

54. Breza-Boruta, B., Kotwica, K. & Bauza-Kaszewska, J. Effect of Tillage System and Organic Matter Management Interactions on Soil Chemical Properties and Biological Activity in a Spring Wheat Short-Time Cultivation. Energies 14, 7451 (2021).

55. Camarotto, C. et al. Conservation agriculture and cover crop practices to regulate water, carbon and nitrogen cycles in the low-lying Venetian plain. CATENA 167, 236–249 (2018).

56. Cao, Q. et al. Eleven-year mulching and tillage practices alter the soil quality and bacterial community composition in Northeast China. Archives of Agronomy and Soil Science 68, 1274–1289 (2022).

57. Carof, M., Tourdonnet, S., Saulas, P., Floch, D. & Roger-Estrade, J. Undersowing wheat with different living mulches in a no-till system. II. Competition for light and nitrogen. Agron. Sustain. Dev. 27, 357–365 (2007).

58. Carvalho, J. L. N. et al. Changes of chemical properties in an oxisol after clearing of native Cerrado vegetation for agricultural use in Vilhena, Rondonia State, Brazil. Soil and Tillage Research 96, 95–102 (2007).

59. Castellanos-Navarrete, A. et al. Earthworm activity and soil structural changes under conservation agriculture in central Mexico. Soil and Tillage Research 123, 61–70 (2012).

60. Castelli, M., Urcoviche, R. C., Gimenes, R. M. T. & Alberton, O. Arbuscular mycorrhizal fungi diversity in maize under different soil managements and seed treatment with fungicide.

61. Ceja-Navarro, J. A. et al. Phylogenetic and Multivariate Analyses To Determine the Effects of Different Tillage and Residue Management Practices on Soil Bacterial Communities. Appl Environ Microbiol 76, 3685–3691 (2010).

62. Chapagain, T. et al. Intercropping of maize, millet, mustard, wheat and ginger increased land productivity and potential economic returns for smallholder terrace farmers in Nepal. Field Crops Research 227, 91–101 (2018).

63. Chávez-Romero, Y. et al. 16S metagenomics reveals changes in the soil bacterial community driven by soil organic C, N-fertilizer and tillage-crop residue management. Soil and Tillage Research 159, 1–8 (2016).

64. Chen, X., Yang, S.-H., Jiang, Z.-W., Ding, J. & Sun, X. Biochar as a tool to reduce environmental impacts of nitrogen loss in water-saving irrigation paddy field. Journal of Cleaner Production 290, 125811 (2021).

65. Chen, X. et al. Long‐term continuous cropping affects ecoenzymatic stoichiometry of microbial nutrient acquisition: a case study from a Chinese Mollisol. J Sci Food Agric 101, 6338–6346 (2021).

66. Chen, Y. et al. Effects of plastic film combined with straw mulch on grain yield and water use efficiency of winter wheat in Loess Plateau. Field Crops Research 172, 53–58 (2015).

67. Chhabra, S. et al. Fertilization management affects the alkaline phosphatase bacterial community in barley rhizosphere soil. Biol Fertil Soils 49, 31–39 (2013).

68. Choudhary, M. et al. Changes in soil biology under conservation agriculture based sustainable intensification of cereal systems in Indo-Gangetic Plains. Geoderma 313, 193–204 (2018).

69. Choudhary, M. et al. Sustainable intensification influences soil quality, biota, and productivity in cereal-based agroecosystems. Applied Soil Ecology 126, 189–198 (2018).

70. Choudhary, M., Jat, H. S., Jat, M. L. & Sharma, P. C. Climate-smart agricultural practices influence the fungal communities and soil properties under major agri-food systems. Front. Microbiol. 13, (2022).

71. Choudhary, M. et al. Soil biological properties and fungal diversity under conservation agriculture in Indo-Gangetic Plains of India. J. Soil Sci. Plant Nutr. 0–0 (2018) doi:10.4067/S0718-95162018005003201.

72. D. McDonald, M. et al. Carbon dioxide mitigation potential of conservation agriculture in a semi-arid agricultural region. AIMS Agriculture and Food 4, 206–222 (2019).

73. Dalal, R. C., Allen, D. E., Wang, W. J., Reeves, S. & Gibson, I. Organic carbon and total nitrogen stocks in a Vertisol following 40 years of no-tillage, crop residue retention and nitrogen fertilisation. Soil and Tillage Research 112, 133–139 (2011).

74. Dalal, R. C., Wang, W., Allen, D. E., Reeves, S. & Menzies, N. W. Soil Nitrogen and Nitrogen-Use Efficiency under Long-Term No-till Practice. Soil Science Society of America Journal 75, 2251–2261 (2011).

75. Dang, T.-H., Cai, G.-X., Guo, S.-L., Hao, M.-D. & Heng, L. K. Effect of Nitrogen Management on Yield and Water Use Efficiency of Rainfed Wheat and Maize in Northwest China. Pedosphere 16, 495–504 (2006).

76. Das, T. K. et al. Impacts of conservation agriculture on total soil organic carbon retention potential under an irrigated agro-ecosystem of the western Indo-Gangetic Plains. European Journal of Agronomy 51, 34–42 (2013).

77. Das, T. K. et al. Conservation agriculture effects on crop and water productivity, profitability and soil organic carbon accumulation under a maize-wheat cropping system in the North-western Indo-Gangetic Plains. Field Crops Research 215, 222–231 (2018).

78. Dawar, K. et al. Maize productivity and soil nutrients variations by the application of vermicompost and biochar. PLOS ONE 17, e0267483 (2022).

79. Dendooven, L. et al. Global warming potential of agricultural systems with contrasting tillage and residue management in the central highlands of Mexico. Agriculture, Ecosystems & Environment 152, 50–58 (2012).

80. Deng, Y., Feng, G., Chen, X. & Zou, C. Arbuscular mycorrhizal fungal colonization is considerable at optimal Olsen-P levels for maximized yields in an intensive wheat-maize cropping system. Field Crops Research 209, 1–9 (2017).

81. Department of Soil Science, Faculty of Agriculture, Kasetsart University, Bangkok 10900, Thailand et al. Biogeochemical Cycling of Carbon and Nitrogen in Rainfed Rice Production Under Conventional and Organic Rice Farming. Environ. Nat. Resour. J. 20, 1–17 (2022).

82. Devkota, M. et al. Combining permanent beds and residue retention with nitrogen fertilization improves crop yields and water productivity in irrigated arid lands under cotton, wheat and maize. Field Crops Research 149, 105–114 (2013).

83. Ding, L.-J., Su, J.-Q., Sun, G.-X., Wu, J.-S. & Wei, W.-X. Increased microbial functional diversity under long-term organic and integrated fertilization in a paddy soil. Appl Microbiol Biotechnol 102, 1969–1982 (2018).

84. Djigal, D., Saj, S., Rabary, B., Blanchart, E. & Villenave, C. Mulch type affects soil biological functioning and crop yield of conservation agriculture systems in a long-term experiment in Madagascar. Soil and Tillage Research 118, 11–21 (2012).

85. Długosz, J., Piotrowska-Długosz, A., Kotwica, K. & Przybyszewska, E. Application of Multi-Component Conditioner with Clinoptilolite and Ascophyllum nodosum Extract for Improving Soil Properties and Zea mays L. Growth and Yield. Agronomy 10, 2005 (2020).

86. Doan, T. T., Henry-des-Tureaux, T., Rumpel, C., Janeau, J.-L. & Jouquet, P. Impact of compost, vermicompost and biochar on soil fertility, maize yield and soil erosion in Northern Vietnam: A three year mesocosm experiment. Science of The Total Environment 514, 147–154 (2015).

87. Dou, F., Wright, A. L. & Hons, F. M. Sensitivity of Labile Soil Organic Carbon to Tillage in Wheat-Based Cropping Systems. Soil Science Society of America Journal 72, 1445–1453 (2008).

88. Du, P. et al. Characterization on the physiological traits of plants and yield formation capacity upon water- and N-saving conditions in wheat (T. aestivum L.). Acta Physiol Plant 43, 48 (2021).

89. Duan, M. et al. Wheat straw and its biochar differently affect soil properties and field-based greenhouse gas emission in a Chernozemic soil. Biol Fertil Soils 56, 1023–1036 (2020).

90. Duan, Y., Shi, X., Li, S., Sun, X. & He, X. Nitrogen Use Efficiency as Affected by Phosphorus and Potassium in Long-Term Rice and Wheat Experiments. Journal of Integrative Agriculture 13, 588–596 (2014).

91. Duan, Y. et al. Nitrogen use efficiency in a wheat–corn cropping system from 15 years of manure and fertilizer applications. Field Crops Research 157, 47–56 (2014).

92. Dutta, A. et al. Impact of long-term residue burning versus retention on soil organic carbon sequestration under a rice-wheat cropping system. Soil and Tillage Research 221, 105421 (2022).

93. Dutta, A. et al. Conventional and Zero Tillage with Residue Management in Rice–Wheat System in the Indo-Gangetic Plains: Impact on Thermal Sensitivity of Soil Organic Carbon Respiration and Enzyme Activity. IJERPH 20, 810 (2023).

94. Dutta, D. et al. Effect of long-term use of organic, inorganic and integrated management practices on carbon sequestration and soil carbon pools in different cropping systems in Tarai region of Kumayun hills. Indian J Agri Sci 88, 523–529 (2018).

95. Eshel, G., Lifschitz, D., Bonfil, D. J. & Sternberg, M. Carbon exchange in rainfed wheat fields: Effects of long-term tillage and fertilization under arid conditions. Agriculture, Ecosystems & Environment 195, 112–119 (2014).

96. EsperschÃ¼tz, J., Gattinger, A., MÃ¤der, P., Schloter, M. & FlieÃŸbach, A. Response of soil microbial biomass and community structures to conventional and organic farming systems under identical crop rotations: Response of soil microbial biomass and community structures. FEMS Microbiology Ecology 61, 26–37 (2007).

97. Essel, E. et al. Evaluation of bacterial and fungal diversity in a long-term spring wheat – field pea rotation field under different tillage practices. Can. J. Soil. Sci. 98, 619–637 (2018).

98. Essel, E. et al. Bacterial and fungal diversity in rhizosphere and bulk soil under different long-term tillage and cereal/legume rotation. Soil and Tillage Research 194, 104302 (2019).

99. Failla, S., Ingrao, C. & Arcidiacono, C. Energy consumption of rainfed durum wheat cultivation in a Mediterranean area using three different soil management systems. Energy 195, 116960 (2020).

100. Farooq, M., Ullah, N., Nadeem, F., Nawaz, A. & Siddique, K. H. M. Sesbania brown manuring improves soil health, productivity, and profitability of post-rice bread wheat and chickpea. Experimental Agriculture 57, 145–162 (2021).

101. Feng, X. et al. Amendment of crop residue in different forms shifted micro-pore system structure and potential functionality of macroaggregates while changed their mass proportion and carbon storage of paddy topsoil. Geoderma 409, 115643 (2022).

102. Flower, K. C., Cordingley, N., Ward, P. R. & Weeks, C. Nitrogen, weed management and economics with cover crops in conservation agriculture in a Mediterranean climate. Field Crops Research 132, 63–75 (2012).

103. Fonte, S. J. & Six, J. Earthworms and litter management contributions to ecosystem services in a tropical agroforestry system. Ecological Applications 20, 1061–1073 (2010).

104. Franzluebbers, A. J., Schomberg, H. H. & Endale, D. M. Surface-soil responses to paraplowing of long-term no-tillage cropland in the Southern Piedmont USA. Soil and Tillage Research 96, 303–315 (2007).

105. Gairhe, J. J., Adhikari, M., Ghimire, D., Khatri-Chhetri, A. & Panday, D. Intervention of Climate-Smart Practices in Wheat under Rice-Wheat Cropping System in Nepal. Climate 9, 19 (2021).

106. Gao, B. et al. The impact of alternative cropping systems on global warming potential, grain yield and groundwater use. Agriculture, Ecosystems & Environment 203, 46–54 (2015).

107. Gao, F. et al. Effects of residue management strategies on greenhouse gases and yield under double cropping of winter wheat and summer maize. Science of The Total Environment 687, 1138–1146 (2019).

108. Gebru, A. A. et al. Implementation of permanent raised beds contributes to increased crop yield and profitability in the northeastern Tigray region, Ethiopia. Ex. Agric. 55, 807–817 (2019).

109. Ghimire, R., Machado, S. & Rhinhart, K. Long-Term Crop Residue and Nitrogen Management Effects on Soil Profile Carbon and Nitrogen in Wheat–Fallow Systems. Agronomy Journal 107, 2230–2240 (2015).

110. Ghimire, R., Norton, J. B., Stahl, P. D. & Norton, U. Soil Microbial Substrate Properties and Microbial Community Responses under Irrigated Organic and Reduced-Tillage Crop and Forage Production Systems. PLOS ONE 9, e103901 (2014).

111. Ghosh, A. et al. Soil enzymes and microbial elemental stoichiometry as bio-indicators of soil quality in diverse cropping systems and nutrient management practices of Indian Vertisols. Applied Soil Ecology 145, 103304 (2020).

112. Ghosh, M., Swain, D. K., Jha, M. K. & Tewari, V. K. Chlorophyll Meter-Based Nitrogen Management in a Rice–Wheat Cropping System in Eastern India. Int. J. Plant Prod. 14, 355–371 (2020).

113. Ghosh, S. et al. Energy budgeting and carbon footprint of contrasting tillage and residue management scenarios in rice-wheat cropping system. Soil and Tillage Research 223, 105445 (2022).

114. Guo, Y. et al. Effects of green manure rotation on soil properties and yield and quality of silage maize in saline-alkali soils. Chinese Journal of Eco-Agriculture, 26, 6, 856-864 (2018).

115. Gong, W., Yan, X., Wang, J., Hu, T. & Gong, Y. Long-term applications of chemical and organic fertilizers on plant-available nitrogen pools and nitrogen management index. Biol Fertil Soils 47, 767–775 (2011).

116. González-Cencerrado, A., Ranz, J. P., López-Franco Jiménez, M. T. & Gajardo, B. R. Assessing the environmental benefit of a new fertilizer based on activated biochar applied to cereal crops. Science of The Total Environment 711, 134668 (2020).

117. Guan, D. et al. Tillage practices effect on root distribution and water use efficiency of winter wheat under rain-fed condition in the North China Plain. Soil and Tillage Research 146, 286–295 (2015).

118. Gupta Choudhury, S. et al. Effect of nutrient management on soil organic carbon sequestration, fertility, and productivity under rice-wheat cropping system in semi-reclaimed sodic soils of North India. Environ Monit Assess 190, 117 (2018).

119. Gupta, R. K. et al. Long-Term Impact of Different Straw Management Practices on Carbon Fractions and Biological Properties under Rice–Wheat System. Agriculture 12, 1733 (2022).

120. Gura, I. & Mnkeni, P. N. S. Crop rotation and residue management effects under no till on the soil quality of a Haplic Cambisol in Alice, Eastern Cape, South Africa. Geoderma 337, 927–934 (2019).

121. Gutiérrez-Núñez, M. S., Gavito, M. E., Ortiz-Salgado, D. & Larsen, J. Agronomic practices and mycorrhizal development and function in maize: Root fungal interactions may affect early nutrition and yield. Rhizosphere 22, 100525 (2022).

122. Guzman, J. G. & Al-Kaisi, M. M. Residue Removal and Management Practices Effects on Soil Environment and Carbon Budget. Soil Science Society of America Journal 78, 609–623 (2014).

123. Gwandu, T. et al. Waste to resource: use of water treatment residual for increased maize productivity and micronutrient content. Environ Geochem Health 44, 3359–3376 (2022).

124. Hai, L., Li, X. G., Li, F. M., Suo, D. R. & Guggenberger, G. Long-term fertilization and manuring effects on physically-separated soil organic matter pools under a wheat–wheat–maize cropping system in an arid region of China. Soil Biology and Biochemistry 42, 253–259 (2010).

125. Haiming, T. et al. Effects of different short-term tillage managements on rhizosphere soil autotrophic CO-fixing bacteria in a double-cropping rice paddy field. Environmental Microbiology Reports 14, 245–253 (2022).

126. Halmi, M. F. A. & Simarani, K. Diazotrophic population and soil nitrogen dynamics following coapplication of biochar with inorganic fertilizer in the humid tropics. Bragantia 80, e3521 (2021).

127. Halvorson, J. J., Liebig, M. A., Archer, D. W., West, M. S. & Tanaka, D. L. Impacts of Crop Sequence and Tillage Management on Soil Carbon Stocks in South-Central North Dakota. Soil Science Society of America Journal 80, 1003–1010 (2016).

128. Harris, R. H., Armstrong, R. D., Wallace, A. J. & Belyaeva, O. N. Effect of nitrogen fertiliser management on soil mineral nitrogen, nitrous oxide losses, yield and nitrogen uptake of wheat growing in waterlogging-prone soils of south-eastern Australia. Soil Res. 54, 619 (2016).

129. Hashimi, R., Matsuura, E. & Komatsuzaki, M. Effects of Cultivating Rice and Wheat with and without Organic Fertilizer Application on Greenhouse Gas Emissions and Soil Quality in Khost, Afghanistan. Sustainability 12, 6508 (2020).

130. Hazarika, S. et al. Effect of tillage system and straw management on organic matter dynamics. Agron. Sustain. Dev. 29, 525–533 (2009).

131. He, L. et al. Biochar mitigated more N-related global warming potential in rice season than that in wheat season: An investigation from ten-year biochar-amended rice-wheat cropping system of China. Science of The Total Environment 821, 153344 (2022).

132. Hok, L. et al. Short-term conservation agriculture and biomass-C input impacts on soil C dynamics in a savanna ecosystem in Cambodia. Agriculture, Ecosystems & Environment 214, 54–67 (2015).

133. Hossain, M. E. et al. Substitution of Chemical Fertilizer with Organic Fertilizer Affects Soil Total Nitrogen and Its Fractions in Northern China. IJERPH 18, 12848 (2021).

134. Hou, P. et al. Effect of long term fertilization management strategies on methane emissions and rice yield. Science of The Total Environment 725, 138261 (2020).

135. Htun, Y. M., Tong, Y., Gao, P. & Xiaotang, J. Coupled effects of straw and nitrogen management on N2O and CH4 emissions of rainfed agriculture in Northwest China. Atmospheric Environment 157, 156–166 (2017).

136. Hua, K., Wang, D., Guo, X. & Guo, Z. Carbon Sequestration Efficiency of Organic Amendments in a Long-Term Experiment on a Vertisol in Huang-Huai-Hai Plain, China. PLOS ONE 9, e108594 (2014).

137. Huang, W. et al. Effects of long-term straw return on soil organic carbon fractions and enzyme activities in a double-cropped rice paddy in South China. Journal of Integrative Agriculture 20, 236–247 (2021).

138. Hurisso, T. T., Norton, J. B. & Norton, U. Soil profile carbon and nitrogen in prairie, perennial grass–legume mixture and wheat-fallow production in the central High Plains, USA. Agriculture, Ecosystems & Environment 181, 179–187 (2013).

139. Iqbal, A. et al. Manure combined with chemical fertilizer increases rice productivity by improving soil health, post-anthesis biomass yield, and nitrogen metabolism. PLoS ONE 15, e0238934 (2020).

140. Islam, K. R. et al. Gypsum, crop rotation, and cover crop impacts on soil organic carbon and biological dynamics in rainfed transitional no-till corn-soybean systems. PLoS ONE 17, e0275198 (2022).

141. Jahangir, M. M. R. et al. Carbon and nitrogen accumulation in soils under conservation agriculture practices decreases with nitrogen application rates. Applied Soil Ecology 168, 104178 (2021).

142. Jamali, H., Quayle, W., Scheer, C. & Baldock, J. Mitigation of N2O emissions from surface-irrigated cropping systems using water management and the nitrification inhibitor DMPP. Soil Res. 54, 481 (2016).

143. Jat, H. S. et al. Climate Smart Agriculture practices improve soil organic carbon pools, biological properties and crop productivity in cereal-based systems of North-West India. CATENA 181, 104059 (2019).

144. Jat, H. S. et al. A Decade of Climate-Smart Agriculture in Major Agri-Food Systems: Earthworm Abundance and Soil Physico-Biochemical Properties. Agronomy 12, 658 (2022).

145. Jat, H. S. et al. A Decade of Climate-Smart Agriculture in Major Agri-Food Systems: Earthworm Abundance and Soil Physico-Biochemical Properties. Agronomy 12, 658 (2022).

146. Jat, R. L. et al. Carbon and nitrogen mineralization in Vertisol as mediated by type and placement method of residue. Environ Monit Assess 190, 439 (2018).

147. Jat, S. L. et al. Dynamics and temperature sensitivity of soil organic carbon mineralization under medium-term conservation agriculture as affected by residue and nitrogen management options. Soil and Tillage Research 190, 175–185 (2019).

148. Jat, S. L. et al. Energy auditing and carbon footprint under long-term conservation agriculture-based intensive maize systems with diverse inorganic nitrogen management options. Science of The Total Environment 664, 659–668 (2019).

149. Jia, G. ‐M., Cao, J. & Wang, G. Influence of land management on soil nutrients and microbial biomass in the central loess plateau, northwest China. Land Degrad Dev 16, 455–462 (2005).

150. Jiménez-Bueno, N. G. et al. Bacterial indicator taxa in soils under different long-term agricultural management. J Appl Microbiol 120, 921–933 (2016).

151. Jin, K. et al. Effects of different soil management practices on winter wheat yield and N losses on a dryland loess soil in China. Soil Res. 46, 455 (2008).

152. Jin, L. et al. Effects of integrated agronomic management practices on yield and nitrogen efficiency of summer maize in North China. Field Crops Research 134, 30–35 (2012).

153. Kanarek, P., Breza-Boruta, B., Bauza-Kaszewska, J. & Lamparski, R. Application of Straw and Biopreparations as a Sustainable Method for Increasing the Organic Carbon Content and Chemical, Physical, and Biological Soil Properties in Spring Barley Culture. Energies 15, 6903 (2022).

154. Karunakaran, V. & Behera, U. K. Tillage and residue management for improving productivity and resource-use efficiency in soybean (glycine max)—wheat (triticum aestivum) cropping system. Ex. Agric. 52, 617–634 (2016).

155. Kayikcioglu, H. H. Can treated wastewater be used as an alternative water resource for agricultural irrigation? changes in soil and plant health after three years of maize cultivation in Western Anatolia, turkey. Appl. Ecol. Env. Res. 16, 8131–8161 (2018).

156. Khan, M. S. A. et al. Complementary effect of zoo compost with mineral nitrogen fertilisation increases wheat yield and nutrition in a low-nutrient soil. Pedosphere 32, 339–347 (2022).

157. Khokhar, A. et al. Soil Properties, Nutrient Availability vis-à-vis Uptake and Productivity of Rainfed Maize-Wheat System in Response to Long-Term Tillage and N Management in Northwest India. Communications in Soil Science and Plant Analysis 53, 2935–2954 (2022).

158. Kihara, J. et al. Crop and Soil Response to Tillage and Crop Residue Application in a Tropical Ferralsol in Sub-humid Western Kenya. in Lessons learned from Long-term Soil Fertility Management Experiments in Africa (eds. Bationo, A. et al.) 41–57 (Springer Netherlands, Dordrecht, 2012). doi:10.1007/978-94-007-2938-4_3.

159. Kim, Y.-N. et al. Co-Responses of Soil Organic Carbon Pool and Biogeochemistry to Different Long-Term Fertilization Practices in Paddy Fields. Plants 11, 3195 (2022).

160. Kukal, S., Rehanarasool & Benbi, D. Soil organic carbon sequestration in relation to organic and inorganic fertilization in rice–wheat and maize–wheat systems. Soil and Tillage Research 102, 87–92 (2009).

161. Kulagowski, R. et al. Effects of conservation agriculture maize-based cropping systems on soil health and crop performance in New Caledonia. Soil and Tillage Research 212, 105079 (2021).

162. Kumar, B. et al. Microbial Biomass Carbon, Activity of Soil Enzymes, Nutrient Availability, Root Growth, and Total Biomass Production in Wheat Cultivars under Variable Irrigation and Nutrient Management. Agronomy 11, 669 (2021).

163. Kumar, R. et al. Impact of crop establishment and residue management on soil properties and productivity in rice-fallow ecosystems in India. Land Degradation & Development 33, 798–812 (2022).

164. Kurniawan, A. H., Sato, S., Cheng, W., Dewi, P. K. & Kobayashi, K. Animal abundance and soil properties affected by long-term organic farming in rice paddies in a typical Japanese yatsuda landscape. Environ Monit Assess 193, 273 (2021).

165. Kushwa, V. et al. Long-term Conservation Tillage Effect on Soil Organic Carbon and Available Phosphorous Content in Vertisols of Central India. Agric Res 5, 353–361 (2016).

166. Kushwaha, C. P., Tripathi, S. K. & Singh, K. P. Variations in soil microbial biomass and N availability due to residue and tillage management in a dryland rice agroecosystem. Soil and Tillage Research 56, 153–166 (2000).

167. Lal, B. et al. Utilization of byproducts of sheep farming as organic fertilizer for improving soil health and productivity of barley forage. Journal of Environmental Management 269, 110765 (2020).

168. Larsen, E. et al. Soil biological properties, soil losses and corn yield in long-term organic and conventional farming systems. Soil and Tillage Research 139, 37–45 (2014).

169. Laudicina, V. A., Novara, A., Barbera, V., Egli, M. & Badalucco, L. Long-Term Tillage and Cropping System Effects on Chemical and Biochemical Characteristics of Soil Organic Matter in a Mediterranean Semiarid Environment. Land Degradation & Development 26, 45–53 (2015).

170. Lavelle, P. et al. Soil ecosystem services and land use in the rapidly changing Orinoco River Basin of Colombia. Agriculture, Ecosystems & Environment 185, 106–117 (2014).

171. Layek, J. et al. Seaweed extract as organic bio-stimulant improves productivity and quality of rice in eastern Himalayas. J Appl Phycol 30, 547–558 (2018).

172. Lee, J. & Choi, H. L. The dynamics of nitrogen derived from a chemical nitrogen fertilizer with treated swine slurry in paddy soil-plant systems. PLoS ONE 12, e0174747 (2017).

173. Lewis, D. B., Kaye, J. P., Jabbour, R. & Barbercheck, M. E. Labile carbon and other soil quality indicators in two tillage systems during transition to organic agriculture. Renew. Agric. Food Syst. 26, 342–353 (2011).

174. Li, C., Yan, K., Tang, L., Jia, Z. & Li, Y. Change in deep soil microbial communities due to long-term fertilization. Soil Biology and Biochemistry 75, 264–272 (2014).

175. Li, H. et al. Variations in soil bacterial taxonomic profiles and putative functions in response to straw incorporation combined with N fertilization during the maize growing season. Agriculture, Ecosystems & Environment 283, 106578 (2019).

176. Li, S. et al. Impact of Straw Return on Soil Carbon Indices, Enzyme Activity, and Grain Production. Soil Science Society of America Journal 81, 1475–1485 (2017).

177. Li, Y.-S. et al. Soil microbial biomass as affected by non-flooded plastic mulching cultivation in rice. Biol Fertil Soils 43, 107–111 (2006).

178. Li, Y. et al. Biochar incorporation increases winter wheat (Triticum aestivum L.) production with significantly improving soil enzyme activities at jointing stage. CATENA 211, 105979 (2022).

179. Li, Z. et al. Effects of straw mulching and nitrogen application rates on crop yields, fertilizer use efficiency, and greenhouse gas emissions of summer maize. Science of The Total Environment 847, 157681 (2022).

180. Li, Z. et al. Evaluation of no-tillage impacts on soil respiration by 13C-isotopic signature in North China Plain. Science of The Total Environment 824, 153852 (2022).

181. Li, Z. et al. In search of long-term sustainable tillage and straw mulching practices for a maize-winter wheat-soybean rotation system in the Loess Plateau of China. Field Crops Research 217, 199–210 (2018).

182. Liang, B., Zhao, W., Yang, X. & Zhou, J. Fate of nitrogen-15 as influenced by soil and nutrient management history in a 19-year wheat–maize experiment. Field Crops Research 144, 126–134 (2013).

183. Lichter, K. et al. Aggregation and C and N contents of soil organic matter fractions in a permanent raised-bed planting system in the Highlands of Central Mexico. Plant Soil 305, 237–252 (2008).

184. Lienhard, P. et al. No-till and cover crops shift soil microbial abundance and diversity in Laos tropical grasslands. Agron. Sustain. Dev. 33, 375–384 (2013).

185. Lin, H. et al. How do soil organic carbon pool, stock and their stability respond to crop residue incorporation in subtropical calcareous agricultural soils? Agriculture, Ecosystems & Environment 332, 107927 (2022).

186. Lin, J. S. et al. Soil organic carbon, aggregation and fungi community after 44 years of no-till and cropping systems in the Central Great Plains, USA. Arch Microbiol 205, 84 (2023).

187. Lin, S. et al. Variations in eco-enzymatic stoichiometric and microbial characteristics in paddy soil as affected by long-term integrated organic-inorganic fertilization. PLoS ONE 12, e0189908 (2017).

188. Liu, B. et al. 14 year applications of chemical fertilizers and crop straw effects on soil labile organic carbon fractions, enzyme activities and microbial community in rice-wheat rotation of middle China. Science of The Total Environment 841, 156608 (2022).

189. Liu, C. et al. Impacts of nitrogen practices on yield, grain quality, and nitrogen-use efficiency of crops and soil fertility in three paddy-upland cropping systems. Journal of the Science of Food and Agriculture 101, 2218–2226 (2021).

190. Liu, E. et al. Long-term effects of no-tillage management practice on soil organic carbon and its fractions in the northern China. Geoderma 213, 379–384 (2014).

191. Liu, J., Dai, J., Wang, Z. & Zhai, B. Effects of fallow or planting wheat (Triticum aestivum L.) and fertilizing P or fertilizing P and N practices on soil carbon and nitrogen in a low-organic-matter soil. Soil Science and Plant Nutrition 62, 263–270 (2016).

192. Liu, K. & Wiatrak, P. Corn production response to tillage and nitrogen application in dry-land environment. Soil and Tillage Research 124, 138–143 (2012).

193. Liu, W. et al. Greenhouse gas emissions, soil quality, and crop productivity from a mono-rice cultivation system as influenced by fallow season straw management. Environ Sci Pollut Res 23, 315–328 (2016).

194. Liu, W. et al. Arbuscular mycorrhizal fungi in soil and roots respond differently to phosphorus inputs in an intensively managed calcareous agricultural soil. Sci Rep 6, 24902 (2016).

195. Liu, X. et al. Effects of nitrogen-enriched biochar on subtropical paddy soil organic carbon pool dynamics. Science of The Total Environment 851, 158322 (2022).

196. Liu, Y. et al. Using PhenoCams to track crop phenology and explain the effects of different cropping systems on yield. Agricultural Systems 195, 103306 (2022).

197. Liu, Z., Chen, Z., Ma, P., Meng, Y. & Zhou, J. Effects of tillage, mulching and N management on yield, water productivity, N uptake and residual soil nitrate in a long-term wheat-summer maize cropping system. Field Crops Research 213, 154–164 (2017).

198. Liu, Z., Rong, Q., Zhou, W. & Liang, G. Effects of inorganic and organic amendment on soil chemical properties, enzyme activities, microbial community and soil quality in yellow clayey soil. PLoS ONE 12, e0172767 (2017).

199. Lobzang Stanzen, Anil Kumar, B.C. Sharma, R. Puniya, & Ashu Sharma. Weed dynamics and productivity under different tillage and weed-management practices in maize (Zea mays)wheat (Triticum aestivum) cropping sequence. IJA 61, 449–454 (2001).

200. Loke, P. F., Kotzé, E. & Du Preez, C. C. Changes in soil organic matter indices following 32 years of different wheat production management practices in semi-arid South Africa. Nutr Cycl Agroecosyst 94, 97–109 (2012).

201. Lopes, L. D. & Fernandes, M. F. Changes in microbial community structure and physiological profile in a kaolinitic tropical soil under different conservation agricultural practices. Applied Soil Ecology 152, 103545 (2020).

202. Lopes, L. D., Fontes Junior, R. C., Pacheco, E. P. & Fernandes, M. F. Shifts in microbial and physicochemical parameters associated with increasing soil quality in a tropical Ultisol under high seasonal variation. Soil and Tillage Research 206, 104819 (2021).

203. Lu, X., Lu, X. & Liao, Y. Conservation tillage increases carbon sequestration of winter wheat-summer maize farmland on Loess Plateau in China. PLOS ONE 13, e0199846 (2018).

204. Lu, X., Lu, X., Tanveer, S. K., Wen, X. & Liao, Y. Effects of tillage management on soil CO2 emission and wheat yield under rain-fed conditions. Soil Res. 54, 38 (2016).

205. Lupwayi, N. Z., Larney, F. J., Blackshaw, R. E., Kanashiro, D. A. & Pearson, D. C. Phospholipid fatty acid biomarkers show positive soil microbial community responses to conservation soil management of irrigated crop rotations. Soil and Tillage Research 168, 1–10 (2017).

206. Lv, S. H. et al. An Opportunity for Regenerative Rice Production: Combining Plastic Film Cover and Plant Biomass Mulch with No-Till Soil Management to Build Soil Carbon, Curb Nitrogen Pollution, and Maintain High-Stable Yield. Agronomy 9, 600 (2019).

207. Ma, B., Wu, T. & Shang, J. On‐farm comparison of variable rates of nitrogen with uniform application to maize on canopy reflectance, soil nitrate, and grain yield. Z. Pflanzenernähr. Bodenk. 177, 216–226 (2014).

208. Ma, G. et al. Bacterial Community Structure and Predicted Function in Wheat Soil From the North China Plain Are Closely Linked With Soil and Plant Characteristics After Seven Years of Irrigation and Nitrogen Application. Front. Microbiol. 11, 506 (2020).

209. Ma, J., Chen, Y., Wang, K., Huang, Y. & Wang, H. Re-utilization of Chinese medicinal herbal residues improved soil fertility and maintained maize yield under chemical fertilizer reduction. Chemosphere 283, 131262 (2021).

210. Ma, Q., Jiang, C., Li, S. & Yu, W. Maize yield and nitrogen-use characteristics were promoted as consistently improved soil fertility: 6-year straw incorporation in Northeast China. Plant Soil Environ. 67, 383–389 (2021).

211. Ma, Q., Yu, W.-T., Jiang, C.-M., Zhou, H. & Xu, Y.-G. The influences of mineral fertilization and crop sequence on sustainability of corn production in northeastern China. Agriculture, Ecosystems & Environment 158, 110–117 (2012).

212. Madar, R. et al. Crop Residue and Potassium Management on Crop and Soil Properties of Maize and Wheat in No-tillage Systems. Communications in Soil Science and Plant Analysis 52, 769–791 (2021).

213. Maharjan, M., Sanaullah, M., Razavi, B. S. & Kuzyakov, Y. Effect of land use and management practices on microbial biomass and enzyme activities in subtropical top-and sub-soils. Applied Soil Ecology 113, 22–28 (2017).

214. Manjaiah, K. M., Voroney, R. P. & Sen, U. Soil organic carbon stocks, storage profile and microbial biomass under different crop management systems in a tropical agricultural ecosystem. Biol Fertil Soils 32, 273–278 (2000).

215. Mapanda, F., Wuta, M., Nyamangara, J. & Rees, R. M. Effects of organic and mineral fertilizer nitrogen on greenhouse gas emissions and plant-captured carbon under maize cropping in Zimbabwe. Plant Soil 343, 67–81 (2011).

216. Margenot, A. J. et al. Can conservation agriculture improve phosphorus (P) availability in weathered soils? Effects of tillage and residue management on soil P status after 9 years in a Kenyan Oxisol. Soil and Tillage Research 166, 157–166 (2017).

217. Margenot, A. J. et al. Biochemical proxies indicate differences in soil C cycling induced by long-term tillage and residue management in a tropical agroecosystem. Plant Soil 420, 315–329 (2017).

218. Martín-Lammerding, D., Tenorio, J. L., Albarrán, M. M., Zambrana, E. & Walter, I. Influence of tillage practices on soil biologically active organic matter content over a growing season under semiarid Mediterranean climate. Span. j. agric. res. 11, 232–243 (2013).

219. Martínez, E., Fuentes, J.-P., Pino, V., Silva, P. & Acevedo, E. Chemical and biological properties as affected by no-tillage and conventional tillage systems in an irrigated Haploxeroll of Central Chile. Soil and Tillage Research 126, 238–245 (2013).

220. Martínez, J. M., Galantini, J. A., Duval, M. E. & López, F. M. Tillage effects on labile pools of soil organic nitrogen in a semi-humid climate of Argentina: A long-term field study. Soil and Tillage Research 169, 71–80 (2017).

221. Mason, H. E., Navabi, A., Frick, B. L., O’Donovan, J. T. & Spaner, D. M. The Weed-Competitive Ability of Canada Western Red Spring Wheat Cultivars Grown under Organic Management. Crop Sci. 47, 1167–1176 (2007).

222. Masto, R. E., Chhonkar, P. K., Singh, D. & Patra, A. K. Alternative soil quality indices for evaluating the effect of intensive cropping, fertilisation and manuring for 31 years in the semi-arid soils of India. Environ Monit Assess 136, 419–435 (2008).

223. Mathers, N. J. & Nash, D. M. Effects of tillage practices on soil and water phosphorus and nitrogen fractions in a Chromosol at Rutherglen in Victoria, Australia. Soil Res. 47, 46 (2009).

224. Matsuoka, T., Asagi, N. & Komatsuzaki, M. Response of weeds and rice yield to Italian ryegrass as a cover crop and planting density in organic farming. Agronomy Journal 114, 689–699 (2022).

225. Mcinga, S., Muzangwa, L., Janhi, K. & Mnkeni, P. N. S. Conservation Agriculture Practices Can Improve Earthworm Species Richness and Abundance in the Semi-Arid Climate of Eastern Cape, South Africa. Agriculture 10, 576 (2020).

226. Meena, B. P. et al. Energy budgeting and carbon footprint in long-term integrated nutrient management modules in a cereal- legume (Zea mays – Cicer arietinum) cropping system. Journal of Cleaner Production 314, 127900 (2021).

227. Mehdi, S. M. SITE-SPECIFIC PHOSPHORUS MANAGEMENT WITH INORGANIC FERTILIZER AND MUNICIPAL SOLID WASTE COMPOSTAPPLICATION IN SALT AFFECTED SOIL. PAKJAS 55, 103–110 (2018).

228. Melero, S. et al. Implementation of chiselling and mouldboard ploughing in soil after 8 years of no-till management in SW, Spain: Effect on soil quality. Soil and Tillage Research 112, 107–113 (2011).

229. Melero, S. et al. Long-term effect of tillage, rotation and nitrogen fertiliser on soil quality in a Mediterranean Vertisol. Soil and Tillage Research 114, 97–107 (2011).

230. Menalled, F. D., Gross, K. L. & Hammond, M. Weed Aboveground and Seedbank Community Responses to Agricultural Management Systems. Ecological Applications 11, 1586–1601 (2001).

231. Midya, A. et al. Crop Establishment Methods and Integrated Nutrient Management Improve: Part I. Crop Performance, Water Productivity and Profitability of Rice (Oryza sativa L.) in the Lower Indo-Gangetic Plain, India. Agronomy 11, 1860 (2021).

232. Mo, F. et al. Fate of photosynthesized carbon as regulated by long–term tillage management in a dryland wheat cropping system. Soil Biology and Biochemistry 138, 107581 (2019).

233. Moharana, P. C., Sharma, B. M., Biswas, D. R., Dwivedi, B. S. & Singh, R. V. Long-term effect of nutrient management on soil fertility and soil organic carbon pools under a 6-year-old pearl millet–wheat cropping system in an Inceptisol of subtropical India. Field Crops Research 136, 32–41 (2012).

234. Monaco, S., Hatch, D. J., Sacco, D., Bertora, C. & Grignani, C. Changes in chemical and biochemical soil properties induced by 11-yr repeated additions of different organic materials in maize-based forage systems. Soil Biology and Biochemistry 40, 608–615 (2008).

235. Mondal, S. et al. Can yield, soil C and aggregation be improved under long-term conservation agriculture in the eastern Indo-Gangetic plain of India? European Journal of Soil Science 72, 1742–1761 (2021).

236. Mondal, S. et al. Effect of conservation tillage and rice-based cropping systems on soil aggregation characteristics and carbon dynamics in Eastern Indo-Gangetic Plain. Paddy Water Environ 18, 573–586 (2020).

237. Muhammad, I. et al. Irrigation and Nitrogen Fertilization Alter Soil Bacterial Communities, Soil Enzyme Activities, and Nutrient Availability in Maize Crop. Front. Microbiol. 13, (2022).

238. Munera-Echeverri, J. L., Martinsen, V., Strand, L. T., Cornelissen, G. & Mulder, J. Effect of conservation farming and biochar addition on soil organic carbon quality, nitrogen mineralization, and crop productivity in a light textured Acrisol in the sub-humid tropics. PLOS ONE 15, e0228717 (2020).

239. Mussarat, M. et al. Comparing the phosphorus use efficiency of pre-treated (organically) rock phosphate with soluble P fertilizers in maize under calcareous soils. PeerJ 9, e11452 (2021).

240. Naab, J. B., Mahama, G. Y., Koo, J., Jones, J. W. & Boote, K. J. Nitrogen and phosphorus fertilization with crop residue retention enhances crop productivity, soil organic carbon, and total soil nitrogen concentrations in sandy-loam soils in Ghana. Nutr Cycl Agroecosyst 102, 33–43 (2015).

241. Nafi, E. et al. Interactive effects of conservation tillage, residue management, and nitrogen fertilizer application on soil properties under maize-cotton rotation system on highly weathered soils of West Africa. Soil and Tillage Research 196, 104473 (2020).

242. Nath, C. P. et al. Impact of variable tillage based residue management and legume based cropping for seven years on enzymes activity, soil quality index and crop productivity in rice ecology. Environmental and Sustainability Indicators 10, 100107 (2021).

243. Nath, C. P. et al. Pulse crop and organic amendments in cropping system improve soil quality in rice ecology: Evidence from a long–term experiment of 16 years. Geoderma 430, 116334 (2023).

244. Nawaz, A., Farooq, M., Lal, R., Rehman, A., & Hafeez-ur-Rehman. Comparison of conventional and conservation rice-wheat systems in Punjab, Pakistan. Soil and Tillage Research 169, 35–43 (2017).

245. Nawaz, A., Farooq, M., Lal, R., Rehman, A., & Hafeez-ur-Rehman. Comparison of conventional and conservation rice-wheat systems in Punjab, Pakistan. Soil and Tillage Research 169, 35–43 (2017).

246. Nayak, M., Swain, D. K. & Sen, R. Strategic valorization of de-oiled microalgal biomass waste as biofertilizer for sustainable and improved agriculture of rice (Oryza sativa L.) crop. Science of The Total Environment 682, 475–484 (2019).

247. Nazir, S. et al. Bioresource Nutrient Recycling in the Rice–Wheat Cropping System: Cornerstone of Organic Agriculture. Plants 10, 2323 (2021).

248. Nguyen-Van-Hung et al. An assessment of irrigated rice production energy efficiency and environmental footprint with in-field and off-field rice straw management practices. Sci Rep 9, 16887 (2019).

249. Nira, R. & Miura, S. Rice yield and soil fertility of an organic paddy system with winter flooding. Soil Science and Plant Nutrition 65, 377–385 (2019).

250. Norkaew, S., Miles, R. J., Brandt, D. K. & Anderson, S. H. Effects of 130 Years of Selected Cropping Management Systems on Soil Health Properties for Sanborn Field. Soil Science Society of America Journal 83, 1479–1490 (2019).

251. Nyambo, Patrick, et al. "Tillage, crop rotation, residue management and biochar influence on soil chemical and biological properties." South African Journal of Plant and Soil 38.5 (2021): 390-397.

252. Obour, A. K., Mikha, M. M., Holman, J. D. & Stahlman, P. W. Changes in soil surface chemistry after fifty years of tillage and nitrogen fertilization. Geoderma 308, 46–53 (2017).

253. Odlare, M., Pell, M. & Svensson, K. Changes in soil chemical and microbiological properties during 4 years of application of various organic residues. Waste Management 28, 1246–1253 (2008).

254. Okeyo, A. I. et al. Effects of selected soil and water conservation technologies on nutrient losses and maize yields in the central highlands of Kenya. Agricultural Water Management 137, 52–58 (2014).

255. Orsini, R., Fiorentini, M. & Zenobi, S. Evaluation of Soil Management Effect on Crop Productivity and Vegetation Indices Accuracy in Mediterranean Cereal-Based Cropping Systems. Sensors 20, 3383 (2020).

256. Verdenilli, R. et al. Influence of 12-years of NPS fertilization on soil quality, microbial community profile and activity under conservation agricultural management. Ciencia del suelo 36, 1 (2018).

257. Oyeogbe, A. I., Das, T. K. & Bandyopadhyay, K. K. Agronomic productivity, nitrogen fertilizer savings and soil organic carbon in conservation agriculture: efficient nitrogen and weed management in maize-wheat system. Archives of Agronomy and Soil Science 64, 1635–1645 (2018).

258. Öztürkmen, A. R. Effects of soil tillage methods on soil quality under corn (Zea mays L.) growth.

259. Zhao, H. L. et al. Effect of straw returning mode on soil organic carbon sequestration. Acta Pedologica Sinica 58, 1, 213-224 (2021).

260. Pan, J. et al. Grain yield, water productivity and nitrogen use efficiency of rice under different water management and fertilizer-N inputs in South China. Agricultural Water Management 184, 191–200 (2017).

261. Pande, K. R. & Becker, M. Seasonal soil nitrogen dynamics in rice‐wheat cropping systems of Nepal. Z. Pflanzenernähr. Bodenk. 166, 499–506 (2003).

262. Paré, M. C., Lafond, J. & Pageau, D. Best management practices in Northern agriculture: A twelve-year rotation and soil tillage study in Saguenay–Lac-Saint-Jean. Soil and Tillage Research 150, 83–92 (2015).

263. Pareja-Sánchez, E., Plaza-Bonilla, D., Álvaro-Fuentes, J. & Cantero-Martínez, C. Is it feasible to reduce tillage and N use while improving maize yield in irrigated Mediterranean agroecosystems? European Journal of Agronomy 109, 125919 (2019).

264. Pareja‐Sánchez, E., Cantero‐Martínez, C., Álvaro‐Fuentes, J. & Plaza‐Bonilla, D. Soil organic carbon sequestration when converting a rainfed cropping system to irrigated corn under different tillage systems and N fertilizer rates. Soil Science Soc of Amer J 84, 1219–1232 (2020).

265. Parihar, C. M. et al. Dependence of temperature sensitivity of soil organic carbon decomposition on nutrient management options under conservation agriculture in a sub-tropical Inceptisol. Soil and Tillage Research 190, 50–60 (2019).

266. Parihar, C. M. et al. Soil quality and carbon sequestration under conservation agriculture with balanced nutrition in intensive cereal-based system. Soil and Tillage Research 202, 104653 (2020).

267. Parihar, C. M. et al. Long term effect of conservation agriculture in maize rotations on total organic carbon, physical and biological properties of a sandy loam soil in north-western Indo-Gangetic Plains. Soil and Tillage Research 161, 116–128 (2016).

268. Parihar, C. M. et al. Long-Term Conservation Agriculture and Intensified Cropping Systems: Effects on Growth, Yield, Water, and Energy-use Efficiency of Maize in Northwestern India. Pedosphere 28, 952–963 (2018).

269. Patra, B., Jena, S. & Phonglosa, A. Date of Planting, Spacing and Integrated Nutrient Management Practices Improves Soil Microbial Populations and Soil Fertility Status Under Rice -Arhar Cropping System in Acidic Soils of Eastern India. Communications in Soil Science and Plant Analysis 54, 1397–1414 (2023).

270. Pattanayak, S. et al. Weed Management and Crop Establishment Methods in Rice (Oryza sativa L.) Influence the Soil Microbial and Enzymatic Activity in Sub-Tropical Environment. Plants 11, 1071 (2022).

271. Paul, B. K. et al. Medium-term impact of tillage and residue management on soil aggregate stability, soil carbon and crop productivity. Agriculture, Ecosystems & Environment 164, 14–22 (2013).

272. Pearsons, K. A., Omondi, E. C., Zinati, G., Smith, A. & Rui, Y. A tale of two systems: Does reducing tillage affect soil health differently in long-term, side-by-side conventional and organic agricultural systems? Soil and Tillage Research 226, 105562 (2023).

273. Peña, D. et al. Using olive mill waste compost with sprinkler irrigation as a strategy to achieve sustainable rice cropping under Mediterranean conditions. Agron. Sustain. Dev. 42, 36 (2022).

274. Plaza-Bonilla, D. et al. Do no-till and pig slurry application improve barley yield and water and nitrogen use efficiencies in rainfed Mediterranean conditions? Field Crops Research 203, 74–85 (2017).

275. Plaza-Bonilla, D., Lampurlanés, J., Fernández, F. G. & Cantero-Martínez, C. Nitrogen fertilization strategies for improved Mediterranean rainfed wheat and barley performance and water and nitrogen use efficiency. European Journal of Agronomy 124, 126238 (2021).

276. Pooniya, V. et al. Six years of conservation agriculture and nutrient management in maize–mustard rotation: Impact on soil properties, system productivity and profitability. Field Crops Research 260, 108002 (2021).

277. Pooniya, V. et al. Conservation agriculture based integrated crop management sustains productivity and economic profitability along with soil properties of the maize-wheat rotation. Sci Rep 12, 1962 (2022).

278. Povilaitis, V. et al. Relationship between spring barley productivity and growing management in Lithuania’s lowland. Acta Agriculturae Scandinavica, Section B — Soil & Plant Science 68, 86–95 (2018).

279. Prasad Datta, S., Kumar Rattan, R. & Chandra, S. Labile soil organic carbon, soil fertility, and crop productivity as influenced by manure and mineral fertilizers in the tropics. Z. Pflanzenernähr. Bodenk. 173, 715–726 (2010).

280. Pu, C. et al. Greenhouse gas emissions from the wheat-maize cropping system under different tillage and crop residue management practices in the North China Plain. Science of The Total Environment 819, 153089 (2022).

281. Purakayastha, T. J. et al. Long-term effects of different land use and soil management on various organic carbon fractions in an Inceptisol of subtropical India. Soil Res. 45, 33 (2007).

283. Puste, A. M., Bandyopadhyay, S. & Das, D. K. Economy of Fertilizer Nitrogen through Organic Sources in Rain-Fed Rice-Legume Cropping Systems in West Bengal, India. The Scientific World JOURNAL 1, 722–727 (2001).

284. Qi, J.-Y. et al. Effects of tillage management on soil carbon decomposition and its relationship with soil chemistry properties in rice paddy fields. Journal of Environmental Management 279, 111595 (2021).

285. Qiu, S. et al. Long-term combined organic manure and chemical fertilizer application enhances aggregate-associated C and N storage in an agricultural Udalfs soil. PLoS ONE 18, e0276197 (2023).

286. Qiu, S. et al. Improved Nitrogen Management for an Intensive Winter Wheat/Summer Maize Double-cropping System. Soil Science Society of America Journal 76, 286–297 (2012).

287. Ramirez-Villanueva, D. A. et al. Bacterial community structure in maize residue amended soil with contrasting management practices. Applied Soil Ecology 90, 49–59 (2015).

288. Ranaivoson, L., Naudin, K., Ripoche, A., Rabeharisoa, L. & Corbeels, M. Effectiveness of conservation agriculture in increasing crop productivity in low-input rainfed rice cropping systems under humid subtropical climate. Field Crops Research 239, 104–113 (2019).

289. Ranva, S. et al. Impact of Safe Rock® Minerals, Mineral Fertilizers, and Manure on the Quantity and Quality of the Wheat Yield in the Rice–Wheat Cropping System. Plants 11, 183 (2022).

290. Rizhiya, E. Y., Mukhina, I. M., Balashov, E. V., Šimansky, V. & Buchkina, N. P. Effect of biochar on N2O emission, crop yield and properties of Stagnic Luvisol in a field experiment. Zemdirbyste-Agriculture 106, 297–306 (2019).

291. Romanos, D., Nemer, N., Khairallah, Y. & Abi Saab, M. T. Application of sewage sludge for cereal production in a Mediterranean environment (Lebanon). IJROWA (2021) doi:10.30486/ijrowa.2021.1903739.1098.

292. Ronanki, S. & Behera, U. K. Effect of conservation agricultural practices and nitrogen management on soil properties. Indian J Agri Sci 89, (2019).

293. Ronanki, S. et al. Effect of conservation agricultural practices and nitrogen management on growth, physiological indices, yield and nutrient uptake of soybean (Glycine max).

294. Rosa, A. T. et al. Contributions of individual cover crop species to rainfed maize production in semi-arid cropping systems. Field Crops Research 271, 108245 (2021).

295. Rosenzweig, S. T., Fonte, S. J. & Schipanski, M. E. Intensifying rotations increases soil carbon, fungi, and aggregation in semi-arid agroecosystems. Agriculture, Ecosystems & Environment 258, 14–22 (2018).

296. Roy, D. et al. Impact of long term conservation agriculture on soil quality under cereal based systems of North West India. Geoderma 405, 115391 (2022).

297. Rusinamhodzi, L., Corbeels, M., Zingore, S., Nyamangara, J. & Giller, K. E. Pushing the envelope? Maize production intensification and the role of cattle manure in recovery of degraded soils in smallholder farming areas of Zimbabwe. Field Crops Research 147, 40–53 (2013).

298. Sadeghi, H. & Jafar Bahrani, M. Effects of Crop Residue and Nitrogen Rates on Yield and Yield Components of Two Dryland Wheat ( Triticum aestivum L.) Cultivars. Plant Production Science 12, 497–502 (2009).

299. Sadiq, M., Li, G., Rahim, N. & Tahir, M. Effect of conservation tillage on yield of spring wheat (Triticum aestivum L.) and soil mineral nitrogen and carbon content. Int. Agrophys. 35, 83–95 (2021).

300. Saha, R. & Ghosh, P. K. Soil Organic Carbon Stock, Moisture Availability and Crop Yield as Influenced by Residue Management and Tillage Practices in Maize–Mustard Cropping System Under Hill Agro-Ecosystem. Natl. Acad. Sci. Lett. 36, 461–468 (2013).

301. Saikia, R., Sharma, S., Thind, H. S., Sidhu, H. S., & Yadvinder-Singh. Temporal changes in biochemical indicators of soil quality in response to tillage, crop residue and green manure management in a rice-wheat system. Ecological Indicators 103, 383–394 (2019).

302. Sainju, U. M., Lenssen, A., Caesar‐Thonthat, T. & Waddell, J. Carbon Sequestration in Dryland Soils and Plant Residue as Influenced by Tillage and Crop Rotation. J of Env Quality 35, 1341–1347 (2006).

303. Sainju, U. M., Lenssen, A. W., Allen, B. L., Stevens, W. B. & Jabro, J. D. Soil total carbon and nitrogen and crop yields after eight years of tillage, crop rotation, and cultural practice. Heliyon 3, e00481 (2017).

304. Sainju, U. M., Lenssen, A. W., Allen, B. L., Stevens, W. B. & Jabro, J. D. Nitrogen balance in dryland agroecosystem in response to tillage, crop rotation, and cultural practice. Nutr Cycl Agroecosyst 110, 467–483 (2018).

305. Sainju, U. M., Stevens, W. B., Caesar-TonThat, T. & Jabro, J. D. Land Use and Management Practices Impact on Plant Biomass Carbon and Soil Carbon Dioxide Emission. Soil Science Soc of Amer J 74, 1613–1622 (2010).

306. Sainju, U. M., Stevens, W. B., Caesar-TonThat, T., Liebig, M. A. & Wang, J. Net Global Warming Potential and Greenhouse Gas Intensity Influenced by Irrigation, Tillage, Crop Rotation, and Nitrogen Fertilization. Journal of Environmental Quality 43, 777–788 (2014).

307. Santillano-Cázares, J., Núñez-Ramírez, F., Ruíz-Alvarado, C., Cárdenas-Castañeda, M. E. & Ortiz-Monasterio, I. Assessment of Fertilizer Management Strategies Aiming to Increase Nitrogen Use Efficiency of Wheat Grown Under Conservation Agriculture. Agronomy 8, 304 (2018).

308. Sarkar, S. & Singh, S. R. Interactive effect of tillage depth and mulch on soil temperature, productivity and water use pattern of rainfed barley (Hordium vulgare L.). Soil and Tillage Research 92, 79–86 (2007).

309. Sarker, J. R. et al. Agricultural management practices impacted carbon and nutrient concentrations in soil aggregates, with minimal influence on aggregate stability and total carbon and nutrient stocks in contrasting soils. Soil and Tillage Research 178, 209–223 (2018).

310. Sarker, M. R. et al. Conservation tillage and residue management improve soil health and crop productivity—Evidence from a rice-maize cropping system in Bangladesh. Front. Environ. Sci. 10, 969819 (2022).

311. Sarker, R. R. et al. Tillage and Residue Management Impact on Microbial and Nematode Abundance Under Diverse Rice-Based Cropping Systems in Calcareous and Non-calcareous Floodplain Soils. J Soil Sci Plant Nutr 23, 2138–2151 (2023).

312. Senthamizhkumaran, V. R. et al. Effect of organic and inorganic nutrients on rice (Oryza sativa var. CO 51) productivity and soil fertility in the Western zone of Tamil Nadu, India. JANS 13, 1488–1498 (2021).

313. Sepat, S., Behera, U. K., Sharma, A. R., Das, T. K. & Bhattacharyya, R. Productivity, Organic Carbon and Residual Soil Fertility of Pigeonpea–Wheat Cropping System Under Varying Tillage and Residue Management. Proc. Natl. Acad. Sci., India, Sect. B Biol. Sci. 84, 561–571 (2014).

314. Shaikh, S. A. et al. Effect of wheat residue incorporation with tillage management on physico-chemical properties of soil and sustainability of maize production. Fresenius Environmental Bulletin 29,.

315. Shang, Z. H., Cao, J. J., Degen, A. A., Zhang, D. W. & Long, R. J. A four year study in a desert land area on the effect of irrigated, cultivated land and abandoned cropland on soil biological, chemical and physical properties. CATENA 175, 1–8 (2019).

316. Sharma, K. L. et al. Effects of Conjunctive Use of Organic and Inorganic Sources of Nutrients on Soil Quality Indicators and Soil Quality Index in Sole Maize, Maize + Soybean, and Sole Soybean Cropping Systems in Hot Semi-arid Tropical Vertisol. Communications in Soil Science and Plant Analysis 45, 2118–2140 (2014).

317. Sharma, R. C. & Banik, P. Sustaining Productivity of Baby Corn–Rice Cropping System and Soil Health through Integrated Nutrient Management. Communications in Soil Science and Plant Analysis 47, 1–10 (2016).

318. Sharma, S. et al. Nitrogen and potassium application effects on productivity, profitability and nutrient use efficiency of irrigated wheat (Triticum aestivum L.). PLoS ONE 17, e0264210 (2022).

319. Sharma, S. et al. Effects of crop residue retention on soil carbon pools after 6 years of rice–wheat cropping system. Environ Earth Sci 78, 296 (2019).

320. Sharma, S., Vashisht, B. B., Singh, P. & Singh, Y. Changes in soil aggregate-associated organic carbon, enzymatic activity, and biological pools under conservation agriculture based practices in rice–wheat system. Biomass Conv. Bioref. 13, 13977–13994 (2023).

321. Shen, X. et al. Dynamics of Soil Organic Carbon and Labile Carbon Fractions in Soil Aggregates Affected by Different Tillage Managements. Sustainability 13, 1541 (2021).

322. Shi, P. & Schulin, R. Erosion-induced losses of carbon, nitrogen, phosphorus and heavy metals from agricultural soils of contrasting organic matter management. Science of The Total Environment 618, 210–218 (2018).

323. Singh, C., Tiwari, S., Gupta, V. K. & Singh, J. S. The effect of rice husk biochar on soil nutrient status, microbial biomass and paddy productivity of nutrient poor agriculture soils. CATENA 171, 485–493 (2018).

324. Singh, C., Tiwari, S., Rai, P. K. & Singh, J. S. Sustainable management of paddy crop residues: effects on methanotrophs diversity and value for soil health restoration. Land Degradation & Development 32, 4121–4131 (2021).

325. Singh, H. & Singh, K. P. Effect of plant residue and fertilizer on grain yield of dryland rice under reduced tillage cultivation. Soil and Tillage Research 34, 115–125 (1995).

326. Singh, P., Singh, R. P. & Ghoshal, N. Influence of Herbicide and Soil Amendments on Soil Nitrogen Dynamics, Microbial Biomass, and Crop Yield in Tropical Dryland Agroecosystems. Soil Science Soc of Amer J 76, 2208–2220 (2012).

327. Singh, R. J. et al. Effect of seven years of nutrient supplementation through organic and inorganic sources on productivity, soil and water conservation, and soil fertility changes of maize-wheat rotation in north-western Indian Himalayas. Agriculture, Ecosystems & Environment 249, 177–186 (2017).

328. Sithole, N. J. & Magwaza, L. S. Long-term changes of soil chemical characteristics and maize yield in no-till conservation agriculture in a semi-arid environment of South Africa. Soil and Tillage Research 194, 104317 (2019).

329. Smith, E. G., Janzen, H. H. & Larney, F. J. Long-term cropping system impact on quality and productivity of a Dark Brown Chernozem in southern Alberta. Can. J. Soil. Sci. 95, 177–186 (2015).

330. Soleymani, A., Shahrajabian, M. H. & Khoshkharam, M. Effect of different fertility systems on fresh forage yield and qualitative traits of forage corn.

331. Somanath Nayak, U.K. Behera, S.L. Meena, & Y.S. Shivay. Influence of tillage practices and phosphorous management on productivity of soybean (Glycine max) and soil properties in soybeanwheat cropping sequence under conservation agriculture. IJA 64, 422–425 (2001).

332. Somasundaram, J. et al. Conservation agriculture effects on soil properties and crop productivity in a semiarid region of India. Soil Res. 57, 187 (2019).

333. Sommer, R., Ryan, J., Masri, S., Singh, M. & Diekmann, J. Effect of shallow tillage, moldboard plowing, straw management and compost addition on soil organic matter and nitrogen in a dryland barley/wheat-vetch rotation. Soil and Tillage Research 115–116, 39–46 (2011).

334. Song, K. et al. Influence of tillage practices and straw incorporation on soil aggregates, organic carbon, and crop yields in a rice-wheat rotation system. Sci Rep 6, 36602 (2016).

335. Srinivasarao, Ch. et al. Soil organic carbon dynamics and crop yields of maize (Zea mays)–black gram (Vigna mungo) rotation-based long term manurial experimental system in semi-arid Vertisols of western India. Trop Ecol 60, 433–446 (2019).

336. Staben, M. L., Bezdicek, D. F., Fauci, M. F. & Smith, J. L. Assessment of Soil Quality in Conservation Reserve Program and Wheat-Fallow Soils. Soil Science Society of America Journal 61, 124–130 (1997).

337. Stevens, W. B., Sainju, U. M., Caesar‐TonThat, T. & Iversen, W. M. Malt Barley Yield and Quality Affected by Irrigation, Tillage, Crop Rotation, and Nitrogen Fertilization. Agronomy Journal 107, 2107–2119 (2015).

338. Sudhakar, P., Sakthivel, V., Manimaran, S., Baradhan, G. & Kumar, S. M. S. Impact of integrated plant nutrient management systems on soil physical properties and productivity enhancement in maize (zea mays l.).

339. Sun, S. et al. Effect of Different N Management Strategies on Soil Nitrate Leaching in a Wheat-Maize Rotation System in the Piedmont Plain of Taihang Mountain. in 2012 International Conference on Biomedical Engineering and Biotechnology 1378–1382 (2012). doi:10.1109/iCBEB.2012.167.

340. Suong, M. et al. Impact of a conservation agriculture system on soil characteristics, rice yield, and root-parasitic nematodes in a Cambodian lowland rice field. Journal of Nematology 51, 1–15 (2019).

341. Syswerda, S. p., Corbin, A. t., Mokma, D. l., Kravchenko, A. n. & Robertson, G. p. Agricultural Management and Soil Carbon Storage in Surface vs. Deep Layers. Soil Science Society of America Journal 75, 92–101 (2011).

342. Tadesse, M. et al. The Effect of Climate-Smart Agriculture on Soil Fertility, Crop Yield, and Soil Carbon in Southern Ethiopia. Sustainability 13, 4515 (2021).

343. Tang, H. et al. Effects of long-term organic matter application on soil carbon accumulation and nitrogen use efficiency in a double-cropping rice field. Environmental Research 213, 113700 (2022).

344. Tang, H. et al. Functional soil organic matter fraction in response to short-term tillage management under the double-cropping rice paddy field in southern of China. Environ Sci Pollut Res 28, 48438–48449 (2021).

345. Tellez-Rio, A. et al. Conservation Agriculture practices reduce the global warming potential of rainfed low N input semi-arid agriculture. European Journal of Agronomy 84, 95–104 (2017).

346. Thierfelder, C. & Wall, P. C. Effects of conservation agriculture on soil quality and productivity in contrasting agro-ecological environments of Zimbabwe. Soil Use and Management 28, 209–220 (2012).

347. Thuithaisong, C. et al. Soil-Quality Indicators for Predicting Sustainable Organic Rice Production. Communications in Soil Science and Plant Analysis 42, 548–568 (2011).

348. Tigga, P. et al. Effect of conservation agriculture on soil organic carbon dynamics and mineral nitrogen under different fertilizer management practices in maize (Zea mays)-wheat (Triticum aestivum) cropping system. Indian J Agri Sci 90, 1568–1574 (2020).

349. Triberti, L. et al. Can mineral and organic fertilization help sequestrate carbon dioxide in cropland? European Journal of Agronomy 29, 13–20 (2008).

350. Valenzuela-Balcázar, I. G., Visconti-Moreno, E. F., Faz, Á. & Acosta, J. A. Soil Organic Carbon Dynamics in Two Rice Cultivation Systems Compared to an Agroforestry Cultivation System. Agronomy 12, 17 (2021).

351. Vezzani, F. M. et al. The importance of plants to development and maintenance of soil structure, microbial communities and ecosystem functions. Soil and Tillage Research 175, 139–149 (2018).

352. Wakelin, S. A. et al. The effects of stubble retention and nitrogen application on soil microbial community structure and functional gene abundance under irrigated maize. FEMS Microbiology Ecology 59, 661–670 (2007).

353. Wang, H. et al. Straw incorporation influences soil organic carbon sequestration, greenhouse gas emission, and crop yields in a Chinese rice (Oryza sativa L.) –wheat (Triticum aestivum L.) cropping system. Soil and Tillage Research 195, 104377 (2019).

354. Wang, J. et al. Increasing basal nitrogen fertilizer rate improves grain yield, quality and 2-acetyl-1-pyrroline in rice under wheat straw returning. Front. Plant Sci. 13, 1099751 (2023).

355. Wang, J. & Sainju, U. M. Soil Carbon and Nitrogen Fractions and Crop Yields Affected by Residue Placement and Crop Types. PLOS ONE 9, e105039 (2014).

356. Wang, K. et al. Responses of environmental and soil enzyme stoichiometric characteristics of wheat cropping system to fertilizer management in rain-fed areas of China. Environ Sci Pollut Res 29, 41520–41533 (2022).

357. Wang, L. & Huang, D. Nitrogen and phosphorus losses by surface runoff and soil microbial communities in a paddy field with different irrigation and fertilization managements. PLOS ONE 16, e0254227 (2021).

358. Wang, S. et al. Effects of Zn, macronutrients, and their interactions through foliar applications on winter wheat grain nutritional quality. PLoS ONE 12, e0181276 (2017).

359. Wang, W., Lai, D. Y. F., Wang, C., Pan, T. & Zeng, C. Effects of rice straw incorporation on active soil organic carbon pools in a subtropical paddy field. Soil and Tillage Research 152, 8–16 (2015).

360. Wang, W., Lai, D. Y. F., Wang, C., Tong, C. & Zeng, C. Effects of inorganic amendments, rice cultivars and cultivation methods on greenhouse gas emissions and rice productivity in a subtropical paddy field. Ecological Engineering 95, 770–778 (2016).

361. Wang, W. et al. Impact of straw management on seasonal soil carbon dioxide emissions, soil water content, and temperature in a semi-arid region of China. Science of The Total Environment 652, 471–482 (2019).

362. Wang, Y. et al. 23-year manure and fertilizer application increases soil organic carbon sequestration of a rice–barley cropping system. Biol Fertil Soils 51, 583–591 (2015).

363. Wang, Y. et al. 23-year manure and fertilizer application increases soil organic carbon sequestration of a rice–barley cropping system. Biol Fertil Soils 51, 583–591 (2015).

364. Wang, Y. et al. Intercropping-driven nitrogen trade-off enhances maize productivity in a long-term experiment. Field Crops Research 287, 108671 (2022).

365. Wang, Y. et al. Effects of cultivation management on the winter wheat grain yield and water utilization efficiency. Sci Rep 9, 12733 (2019).

366. Wei, T. et al. Effects of Wheat Straw Incorporation on the Availability of Soil Nutrients and Enzyme Activities in Semiarid Areas. PLoS ONE 10, e0120994 (2015).

367. Werner, S. et al. Agronomic benefits of biochar as a soil amendment after its use as waste water filtration medium. Environmental Pollution 233, 561–568 (2018).

368. Widmer, F., Rasche, F., Hartmann, M. & Fliessbach, A. Community structures and substrate utilization of bacteria in soils from organic and conventional farming systems of the DOK long-term field experiment. Applied Soil Ecology 33, 294–307 (2006).

369. Xu, A. et al. Long-term nitrogen addition impact on agronomic traits, nitrogen uptake and nitrogen resorption efficiency of wheat in a rainfed region. Soil Science Society of America Journal 85, 452–467 (2021).

370. Xu, N., Wilson, H. F., Saiers, J. E. & Entz, M. Effects of Crop Rotation and Management System on Water-Extractable Organic Matter Concentration, Structure, and Bioavailability in a Chernozemic Agricultural Soil. Journal of Environmental Quality 42, 179–190 (2013).

371. Xue, L., Yu, Y. & Yang, L. Maintaining yields and reducing nitrogen loss in rice–wheat rotation system in Taihu Lake region with proper fertilizer management. Environ. Res. Lett. 9, 115010 (2014).

372. Yadav, M. R. et al. Energy-Carbon Footprint, Productivity and Profitability of Barley Cultivars under Contrasting Tillage-Residue Managements in Semi-Arid Plains of North-West India. J Soil Sci Plant Nutr 23, 1109–1124 (2023).

373. Yan, J. et al. Abundance and Diversity of Soybean-Nodulating Rhizobia in Black Soil Are Impacted by Land Use and Crop Management. Applied and Environmental Microbiology 80, 5394–5402 (2014).

374. Yan, J., Wu, Q., Qi, D. & Zhu, J. Rice yield, water productivity, and nitrogen use efficiency responses to nitrogen management strategies under supplementary irrigation for rain-fed rice cultivation. Agricultural Water Management 263, 107486 (2022).

375. Yan, J. et al. Initial Studies on the Effect of the Rice–Duck–Crayfish Ecological Co-Culture System on Physical, Chemical, and Microbiological Properties of Soils: A Field Case Study in Chaohu Lake Basin, Southeast China. International Journal of Environmental Research and Public Health 20, 2006 (2023).

376. Yang, Q., Wang, X., Shen, Y. & Philp, J. N. M. Functional diversity of soil microbial communities in response to tillage and crop residue retention in an eroded Loess soil. Soil Science and Plant Nutrition 59, 311–321 (2013).

377. Yang, S. et al. Effects of Biochar Application on Soil Organic Carbon Composition and Enzyme Activity in Paddy Soil under Water-Saving Irrigation. IJERPH 17, 333 (2020).

378. Yang, S., Sun, X., Ding, J., Jiang, Z. & Xu, J. Effects of biochar addition on the NEE and soil organic carbon content of paddy fields under water-saving irrigation. Environ Sci Pollut Res 26, 8303–8311 (2019).

379. Yang, S., Wang, Y., Liu, R., Xing, L. & Yang, Z. Improved crop yield and reduced nitrate nitrogen leaching with straw return in a rice-wheat rotation of Ningxia irrigation district. Sci Rep 8, 9458 (2018).

380. Yang, X. et al. Combining Organic Fertilizer With Controlled-Release Urea to Reduce Nitrogen Leaching and Promote Wheat Yields. Front. Plant Sci. 12, 802137 (2021).

381. Yang, X., Ren, W., Sun, B. & Zhang, S. Effects of contrasting soil management regimes on total and labile soil organic carbon fractions in a loess soil in China. Geoderma 177–178, 49–56 (2012).

382. Yang, Y., Tong, Y., Liu, G., Han, W. & Li, H. Conservation tillage methods affect soil water use and spring maize yield in a semi-humid drought-prone area of China. Acta Ecologica Sinica 42, 453–460 (2022).

383. Yao, Z. et al. Nitrous oxide and methane fluxes from a rice–wheat crop rotation under wheat residue incorporation and no-tillage practices. Atmospheric Environment 79, 641–649 (2013).

384. Yin, M., Li, Y. & Xu, Y. Comparative effects of nitrogen application on growth and nitrogen use in a winter wheat/summer maize rotation system. Journal of Integrative Agriculture 16, 2062–2072 (2017).

385. Yu, K. et al. Low greenhouse gases emissions associated with high nitrogen use efficiency under optimized fertilization regimes in double-rice cropping systems. Applied Soil Ecology 160, 103846 (2021).

386. Yuan, H. et al. Soil microbial biomass and bacterial and fungal community structures responses to long-term fertilization in paddy soils. J Soils Sediments 13, 877–886 (2013).

387. Yuan, L. et al. Effects of continuous straw returning on bacterial community structure and enzyme activities in rape-rice soil aggregates. Sci Rep 13, 2357 (2023).

388. Zaki, M. K., Komariah, K., Rahmat, A. & Pujiasmanto, B. Organic Amendment and Fertilizer Effect on Soil Chemical Properties and Yield of Maize (Zea mays L.) in Rainfed Condition. Walailak J Sci & Tech 17, 11–17 (2018).

389. Zanatta, J. A., Vieira, F. C. B., Briedis, C., Dieckow, J. & Bayer, C. Carbon indices to assess quality of management systems in a Subtropical Acrisol. Sci. agric. (Piracicaba, Braz.) 76, 501–508 (2019).

390. Zhang, H. et al. Responses of Soil Bacterial and Fungal Communities to Organic and Conventional Farming Systems in East China. Journal of Microbiology and Biotechnology 29, 441–453 (2019).

391. Zhang, H., Zhinfluenceang, Y., Yan, C., Liu, E. & Chen, B. Soil nitrogen and its fractions between long-term conventional and no-tillage systems with straw retention in dryland farming in northern China. Geoderma 269, 138–144 (2016).

392. Zhang, H. et al. Responses of soil organic carbon and crop yields to 33-year mineral fertilizer and straw additions under different tillage systems. Soil and Tillage Research 209, 104943 (2021).

393. Zhang, J. et al. Reduced basal and increased topdressing fertilizer rate combined with straw incorporation improves rice yield stability and soil organic carbon sequestration in a rice–wheat system. Front. Plant Sci. 13, 964957 (2022).

394. Zhang, M., Tian, Y., Zhao, M., Yin, B. & Zhu, Z. The assessment of nitrate leaching in a rice–wheat rotation system using an improved agronomic practice aimed to increase rice crop yields. Agriculture, Ecosystems & Environment 241, 100–109 (2017).

395. Zhang, Q. et al. Effects of six-year biochar amendment on soil aggregation, crop growth, and nitrogen and phosphorus use efficiencies in a rice-wheat rotation. Journal of Cleaner Production 242, 118435 (2020).

396. Zhang, S. et al. Controlled-release urea reduced nitrogen leaching and improved nitrogen use efficiency and yield of direct-seeded rice. Journal of Environmental Management 220, 191–197 (2018).

397. Zhang, S. et al. Long-term manure amendments and chemical fertilizers enhanced soil organic carbon sequestration in a wheat (Triticum aestivum L.)–maize (Zea mays L.) rotation system. Journal of the Science of Food and Agriculture 97, 2575–2581 (2017).

398. Zhang, X. et al. Tillage and residue management for long-term wheat-maize cropping in the North China Plain: I. Crop yield and integrated soil fertility index. oyeiField Crops Research 221, 157–165 (2018).

399. Zhang, X. et al. Effects of tillage and residue management on soil nematode communities in North China. Ecological Indicators 13, 75–81 (2012).

400. Zhang, Y. et al. Effects of different sub-soiling frequencies incorporated into no-tillage systems on soil properties and crop yield in dryland wheat-maize rotation system. Field Crops Research 209, 151–158 (2017).

401. Zhang, Y. et al. Maize yield and soil fertility with combined use of compost and inorganic fertilizers on a calcareous soil on the North China Plain. Soil and Tillage Research 155, 85–94 (2016).

402. Zhang, Z. et al. Do microorganism stoichiometric alterations affect carbon sequestration in paddy soil subjected to phosphorus input? Ecological Applications 25, 866–879 (2015).

403. Zhao, J. et al. A 2-year study on the effects of tillage and straw management on the soil quality and peanut yield in a wheat–peanut rotation system. J Soils Sediments 21, 1698–1712 (2021).

404. Zheng, B. et al. Soil Organic Matter, Aggregates, and Microbial Characteristics of Intercropping Soybean under Straw Incorporation and N Input. Agriculture 12, 1409 (2022).

405. Zhou, J. et al. Influence of 34-years of fertilization on bacterial communities in an intensively cultivated black soil in northeast China. Soil Biology and Biochemistry 90, 42–51 (2015).

406. Zhou, L. et al. Bentonite-humic acid improves soil organic carbon, microbial biomass, enzyme activities and grain quality in a sandy soil cropped to maize (Zea mays L.) in a semi-arid region. Journal of Integrative Agriculture 21, 208–221 (2022).

407. Zhou, M. et al. N2O and CH4 Emissions, and NO3− Leaching on a Crop-Yield Basis from a Subtropical Rain-fed Wheat–Maize Rotation in Response to Different Types of Nitrogen Fertilizer. Ecosystems 17, 286–301 (2014).

408. Zhou, M., Zhu, B., Wang, X. & Wang, Y. Long-term field measurements of annual methane and nitrous oxide emissions from a Chinese subtropical wheat-rice rotation system. Soil Biology and Biochemistry 115, 21–34 (2017).

409. Zhu, L., Hu, N., Yang, M., Zhan, X. & Zhang, Z. Effects of Different Tillage and Straw Return on Soil Organic Carbon in a Rice-Wheat Rotation System. PLoS ONE 9, e88900 (2014).

410. Zhu, X. et al. Microorganisms, their residues, and soil carbon storage under a continuous maize cropping system with eight years of variable residue retention. Applied Soil Ecology 187, 104846 (2023).

411. Zhu, Y. et al. Plastic film mulching improved rhizosphere microbes and yield of rainfed spring wheat. Agricultural and Forest Meteorology 263, 130–136 (2018).

**References** (for Supporting Information only)

1. Zou, Y., Liu, Z., Chen, Y., Wang, Y. & Feng, S. Crop Rotation and Diversification in China: Enhancing Sustainable Agriculture and Resilience. *Agriculture* **14**, 1465 (2024).
2. Diacono, M., Baldivieso-Freitas, P. & Sans Serra, F. X. Nitrogen Utilization in a Cereal-Legume Rotation Managed with Sustainable Agricultural Practices. *Agronomy* **9**, 113 (2019).
3. Wang, X., Qi, J. & Kan, Z. Sustainable Management and Tillage Practice in Agriculture. *Agronomy* **14**, 2891 (2024).
4. Verma, B. C., Pramanik, P. & Bhaduri, D. Organic Fertilizers for Sustainable Soil and Environmental Management. in Nutrient Dynamics for Sustainable Crop Production (ed. Meena, R. S.) 289–313 (Springer, Singapore, 2020). doi:10.1007/978-981-13-8660-2_10.
5. Parven, A., Meftaul, I. M., Venkateswarlu, K. & Megharaj, M. Herbicides in modern sustainable agriculture: environmental fate, ecological implications, and human health concerns. *Int. J. Environ. Sci. Technol*. **22**, 1181–1202 (2025).
6. Quintarelli, V. *et al.* Cover Crops for Sustainable Cropping Systems: A Review. *Agriculture* **12**, 2076 (2022).
7. Maurya, R., Bharti, C., Singh, T. D. & Pratap, V. Crop Residue Management for Sustainable Agriculture. *Int.J.Curr.Microbiol.App.Sci* **9**, 3168–3174 (2020).
8. Chartzoulakis, K. & Bertaki, M. Sustainable Water Management in Agriculture under Climate Change. *Agriculture and Agricultural Science Procedia* **4**, 88–98 (2015).
